# Synthesis of *N,N′*-alkylidene bisamides and Suzuki–Miyaura coupling reaction derivatives with Pd organometallic catalyst anchored to channels of mesoporous silica MCM-41

**DOI:** 10.1038/s41598-024-58310-5

**Published:** 2024-04-02

**Authors:** Sulieman Ibraheem Shelash Al-Hawary, Raed Obaid Saleh, Ahmed Rafiq AlBajalan, Normurot Fayzullaev, Mohammed Alshuhri, Saad Hayif Jasim Ali, Ahmed Alawadi, Mohammed Abed Jawad, Salim B. Alsaadi, Maryam Sadat Ghorayshi Nejad

**Affiliations:** 1 Department of Business Administration, Business School, Al Al-Bayt University, P.O. BOX 130040, Mafraq, 25113 Jordan; 2 Department of Medical Laboratory Techniques, Al-Maarif University College, Al-Anbar, Iraq; 3 Petroleum Technology Department, Erbil Polytechnic University, Erbil, Iraq; 4 Department of Polymer Chemistry and Chemical Technology, Samarkand State University, 140101 Samarkand, Uzbekistan; 5 Radiology and Medical Imaging Department, College of Applied Medical Sciences, Prince Sattam Bin Abdulaziz University, 11942 Kharj, Saudi Arabia; 6 Department of Medical Laboratory, College of Health and Medical Technololgy, Al-Ayen University, Thi-Qar, Iraq; 7 College of Technical Engineering, The Islamic University, Najaf, Iraq; 8 College of Technical Engineering, The Islamic University of Al Diwaniyah, Al Diwaniyah, Iraq; 9 College of Technical Engineering, The Islamic University of Babylon, Babylon, Iraq; 10 Department of Pharmaceutics, Al-Nisour University College, Baghdad, Iraq; 11 Department of Pharmaceutics, Al-Hadi University College, Baghdad, 10011 Iraq; 12 Takin Shimi Sepanta Industries Co, Sirvan Industrial Zone, Ilam, 6958140120 Iran

**Keywords:** Pd-DPyE@MCM-41@MNP, Magnetic organometallic catalyst, Suzuki–Miyaura coupling (SMC) reaction, *N,N*′-alkylidene bisamides, Chemistry, Catalysis, Green chemistry

## Abstract

At first, an organometallic catalyst namely, Pd-DPyE@MCM-41@MNP was prepared through magnetic (Fe_3_O_4_) nanoparticles-doped into channels of mesoporous silica MCM-41 and then, anchoring a novel complex composed of di(4-pyridyl)ethylene and palladium on the inner surface of the support. This immobilized catalyst was successfully identified via VSM, ICP-OES, TEM, FTIR, TGA, SEM, BET, XRD, EDX and elemental mapping analyses. After that, it was used as a versatile, heterogeneous, and magnetically reproducible catalyst in the generation of *N,N*′-alkylidene bisamides (**1a-13a**, 8–20 min, 90–98%, 50 °C, solvent-free) and Suzuki–Miyaura coupling (SMC) reaction derivatives (**1b-26b**, 10–140 min, 86–98%, 60 °C, PEG-400). The VSM plot of Pd-DPyE@MCM-41@MNP displays that this nanocatalyst can be easily recycled by applying an external magnetic field. In both synthetic paths, this nanocatalyst was reused at least seven times without palladium leaching and significantly reducing its catalytic performance. Also, stability and heterogeneous nature of catalyst were approved via ICP-OES technique and hot filtration test.

## Introduction

Nanocatalysts have gained significant attention in organic reactions due to their unique properties and enhanced catalytic performance compared to traditional catalysts^1–3^. They offer various applications and advantages in organic reactions, including increased activity, selectivity, and efficiency^1–3^. Their unique properties and versatility make them promising candidates for advancing various branches of organic chemistry and enabling more sustainable and efficient chemical processes^1–3^.

In recent years, organometallic catalysis has become a powerful tool in various chemical transformations^3,4^. In organometallic catalysis, the metal centers in the catalyst are crucial role in facilitating and controlling chemical reactions^3,4^. These metal centers can be transition metals like Pd, Pt, Ni, or non-transition metals such as Mg, Zn, etc. The organic ligands attached to the metal in the catalyst also contribute to its reactivity and selectivity^5–8^. Organometallic catalysis finds applications in various fields, including pharmaceuticals, agrochemicals, materials science, and more^9–12^. It enables the synthesis of complex and structurally diverse compounds that may be challenging to access through traditional organic synthesis methods. The use of support materials, such as MCM-41, is crucial in preparing organometallic catalysts. Using a support material like MCM-41 helps to enhance the stability of the catalyst^13–15^. The support acts as a protective matrix, preventing the aggregation or decomposition of the active metal species^16^. This stability allows for extended catalyst lifetimes and better control over catalytic reactions. In the realm of catalysis, the concept of a support material acting as a protective matrix refers to how the support physically surrounds and stabilizes the active metal species, shielding it from adverse conditions and interactions that could degrade its performance. This protective matrix helps maintain the integrity and activity of the catalyst. Zeolites, activated carbon, metal–organic frameworks (MOFs), silica supports, alumina supports, and MCM-41 are examples of supports acting as protective matrices in catalysis. In these examples, the supports function as protective matrices by encapsulating, stabilizing, and preventing the aggregation or degradation of active metal species, ultimately contributing to the efficiency and durability of the catalyst system^17–23^.

MCM-41 and other mesoporous supports have a high surface area and well-defined porous structure^24–26^. This feature provides many accessible active sites for the metal catalyst, increasing the overall catalytic activity. The high surface area also allows for better dispersion of metal complexes, ensuring greater reactant exposure and improving reaction rates. The incorporation of magnetic components into MCM-41 nanoparticles adds magnetic properties to the material, enabling the particles to respond to external magnetic fields^16,24–26^.

Organometallic catalysts are widely used in various organic transformations, including the generation of carbon–carbon, carbon–nitrogen, carbon–oxygen, and carbon–sulfur bonds^16^. The remarkable development in this field is due to the importance of such links in the framework of important pharmaceutical, chemical and industrial compounds^16,27–30^. In these transformations, palladium is a multipurpose catalyst, and its vital role in all kinds of coupling reactions, oxidation and reduction and multi-component or domino reactions is mentioned in the literature^16,27–30^. In this work, with the help of a palladium-based catalytic system, the pseudo three-component synthesis of *N,N′*-alkylidene bisamides and Suzuki–Miyaura Coupling (SMC) reaction derivatives has been investigated. *N,N*′-alkylidene bisamides, mainly produced via the condensation of aryl aldehydes (1 mmol) with primary amides (2 mmol), are versatile compounds with considerable potential in various fields^31–37^. Their diverse biological activities and synthetic accessibility make them attractive targets for medicinal and organic chemists, since they can exhibit diverse biological activities, including antimicrobial, antitumor, anti-inflammatory, and enzyme-inhibitory properties. Their activity is often linked to their ability to interact with specific protein targets, modulating biochemical pathways and cellular processes^38–44^. Besides, the SMC reaction is one of the most efficient and general organic transformations for producing herbicides, drugs, liquid crystals, polymers, catalyst ligands, natural products, and modern materials^45,46^. Conventionally, carbon–carbon coupling is performed in the presence of transition metal complexes comprising organic ligands in organic solvents at high temperatures^47^. Nevertheless, there are several problems on the way to produce these SMC derivatives. First and foremost, the organic solvents used may cause irreparable environmental challenges due to their noxiousness, and reactions performed under harsh temperature conditions certainly consume more energy^48^. In the second place, the most frequent catalytic systems are promoted for the carbon–carbon coupling reaction with homogeneous palladium complexes with ligands e.g., carbenes, dibenzylideneacetone, and phosphine, which are expensive to prepare and sensitive to humidity and air. Furthermore, reusing catalysts with such ligands is difficult and has harmful environmental consequences^49,50^. Long reaction times, use of high amounts of metal precursors, difficult separation and purification of products, low yields of products, hard work-up and lack of proper reproducibility of the catalysts used are other problems in this field^51–59^.

According to the above points, in this work, an organometallic catalyst based on palladium supported in the mesoporous channels of MCM-41 magnetic (Fe_3_O_4_) nanoparticles named Pd-DPyE@MCM-41@MNP was designed, produced, and identified. It was used for the rapid production of *N,N*′*-*alkylidene bisamides through the reaction of 1 mmol aldehydes with 2 mmol primary amides, and also the synthesis of SMC derivatives via the reaction of 1 mmol (hetero)aryl halides with 1 mmol benzeneboronic acid under eco-friendly and moderate conditions.

## Experimental

### Materials and devices

Explanations related to the specifications of raw materials and devices are provided in the supplementary material file.

### Production of the nanocatalyst

At first, MCM-41@MNP substrates were produced through doping magnetic (Fe_3_O_4_) nanoparticles (MNPs) into the mesoporous channels of silica. To this end, nude MNPs were produced by chemical co-precipitation approach in a basic solution of Fe^3+^ and Fe^2+^ ions at 80 °C according to the procedure mentioned in the literature^60^. MNPs (2 g) were dispersed in 30 mL H_2_O via ultrasonication within 30 min, and then 150 mL EtOH was added to the resulting mixture and stirred at room ambient. Next, PEG (5.36 g), TEOS (2 mL) and 10 mL of ammonia solution (28 wt%) were added to the resulting suspension with stirring, and it was stirred at ambient temperature for 30 h until NMPs were coated with MCM-41. The acquired MCM-41@MNP was cleaned with distilled H_2_O and EtOH, and it was dried at 25 °C. Subsequently, MCM-41@MNP in a mixture of 30 mL deionized water, 20 mL ethanol, 0.15 g CTAB, and 0.6 mL ammonia solution was dispersed, and stirred for 30 min at 25 °C. Following, 0.4 g TEOS was slowly added to the resulting mixture and stirring was continued for another 5 h. The residual solids were cleaned with deionized water and ethanol, and dried at 80 °C under vacuum conditions (10 h). To eliminate the CTAB template, 5 mL hydrochloric acid (2 mol/L) and 100 mL EtOH were added to the acquired composite (0.1 g) and the resulting mixture was stirred at 25 °C for 24 h. The remaining products (i.e., MCM-41@MNP) were cleaned by a mixture of H_2_O and EtOH and separated via magnetic decantation.

**Scheme 1:**
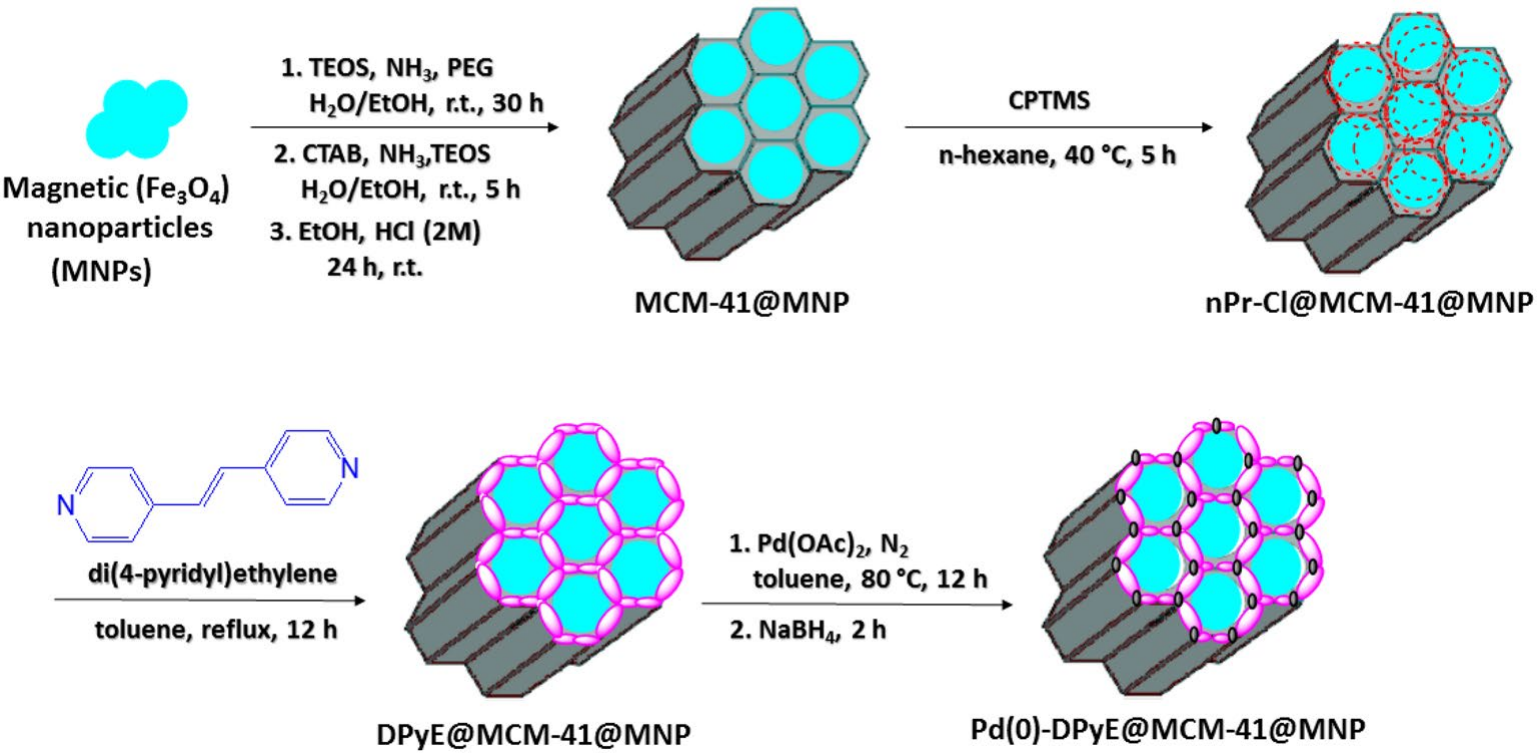
Production steps Pd-DPyE@MCM-41@MNP.

The achieved MCM-41@MNP (1.5 g) was dispersed in 40 mL n-hexane via ultrasonication, then 2.5 mL CPTMS ((3-chloropropyl)trimethoxysilane) was added to this mixture, and stirred for 5 h in N_2_ at 40 °C. Then, the acquired nPr-Cl@MCM-41@MNP was cleaned with EtOH (4 × 5 mL), and isolated by magnetic decantation. At 50 °C, nPr-Cl@MCM-41@MNP was dried. At the next stage, 1 g of nPr-Cl@MCM-41@MNP was dispersed in 50 mL toluene through sonication for 20 min, and 2.5 mmol di(4-pyridyl)ethylene was added to the reaction mixture and stirred under reflux conditions in N_2_ for 12 h. Then, the acquired nanoparticles (DPyE@MCM-41@MNP) were cleaned with EtOH (3 × 5 mL) and dried at 50 °C after separation by magnetic decantation. The produced DPyE@MCM-41@MNP (1 g) in 25 mL toluene was dispersed using sonication (30 min), and 0.50 mmol Pd(OAc)_2_ was added to the mixture, and then stirred for 12 h at 80 °C. Next, the 0.6 mmol NaBH_4_ was added to the obtained mixture and stirred (2 h). In the last step, the final product (Pd(0)-DPyE@MCM-41@MNP) was detached via magnetic decantation, washed several times with ethanol and dried at ambient temperature (Scheme 1).

### General path for the synthesis of *N,N′*-alkylidene bisamides catalyzed by Pd-DPyE@MCM-41@MNP

In the absence of a solvent at a temperature of 50 °C, a mixture containing 10 mg Pd-DPyE@MCM-41@MNP (2.5 mol%), 2 equivalent amide and 1 equivalent aldehyde were heated till the reaction was finished via TLC monitoring. Upon completion of the reaction, the cooled reaction mixture was diluted with 10 mL of ethanol, and the catalyst was isolated via simple filtration technique along with the utilization of an external magnetic field and was cleaned by ethanol (2 × 5 mL) and ethyl acetate (2 × 5 mL), and dried for use in the next run. Finally, to give pure products, the solvent was evaporated and the residue was recrystallized in ethanol (95%).

### General route for SMC reaction catalyzed by Pd-DPyE@MCM-41@MNP

In a flask at a temperature of 60 °C in PEG-400 solvent (2 mL), a mixture of 6 mg Pd-DPyE@MCM-41@MNP (1.50 mol%), 1 equivalent aryl halide (or heteroaryl halide), 1 equivalent benzeneboronic acid and 3 equivalents sodium carbonate were stirred till the reaction was finished via TLC monitoring. Upon completion of the reaction, the catalyst was recovered by simple filtration technique along with the application of an external magnetic field. It was cleaned with ethanol (2 × 5 mL) and ethyl acetate (2 × 5 mL), and dried for employ in the next cycle. Then, the reaction mixture using diethyl ether and water (3 × 5 mL) was extracted and, organic layer with 1.5 g of Na_2_SO_4_ was dried. Finally, derivatives of SMC reaction were generated with suitable efficiency after evaporation of the solvent.

### Ethics approval and consent to participate

The author’s declare that the paper is not be submitted simultaneously to another journal. The submitted work is original and has not been published elsewhere in any form or language, and the authors have no conflict of interest regarding this manuscript. The authors agree to participate in submitting our manuscript to this journal, and agree to the publication of our research data in this journal.

## Results and discussion

**Figure 1:**
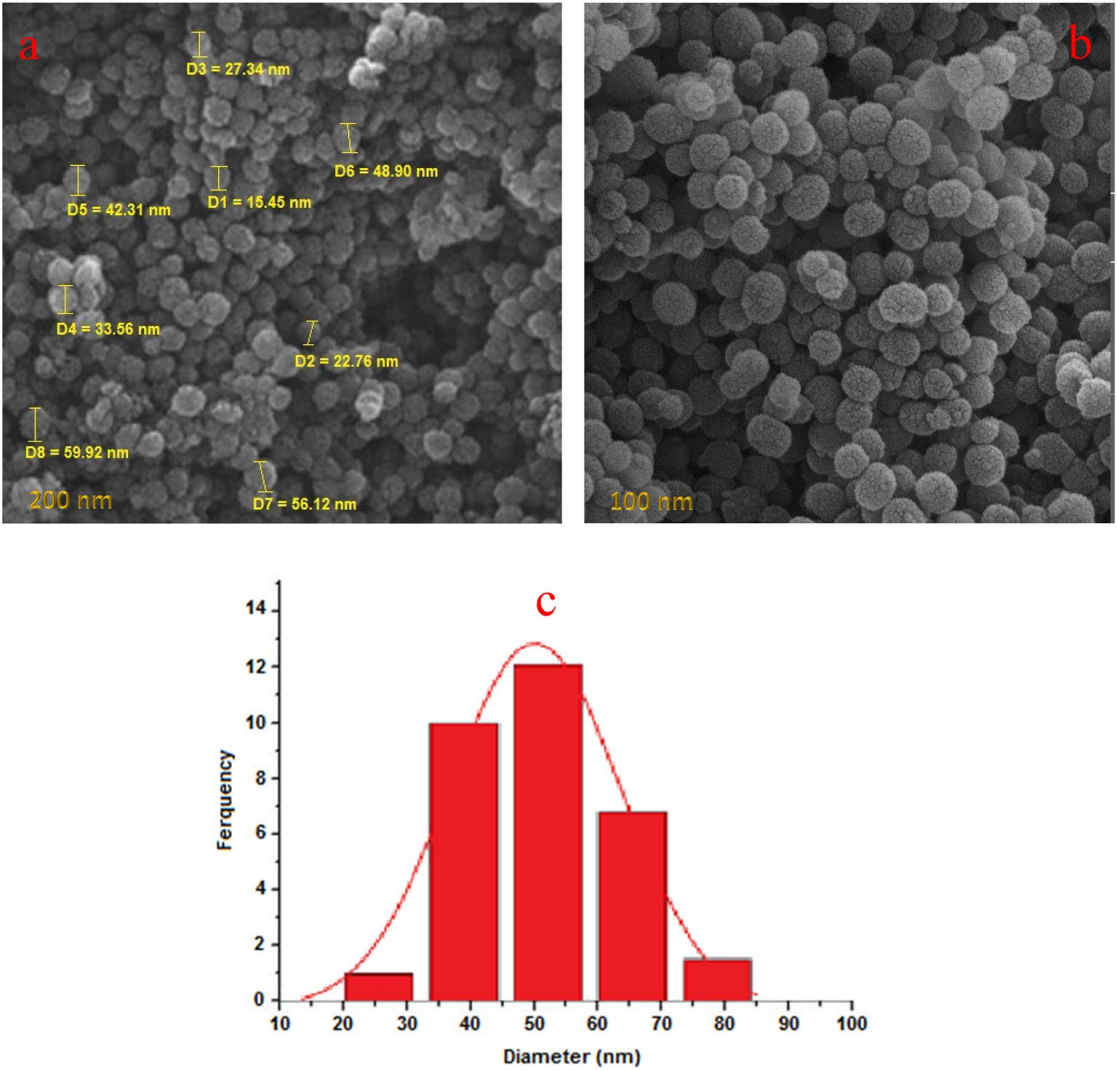
The FE-SEM images (**a**, **b**) and particle size distribution (**c**) of Pd-DPyE@MCM-41@MNP.

**Figure 2:**
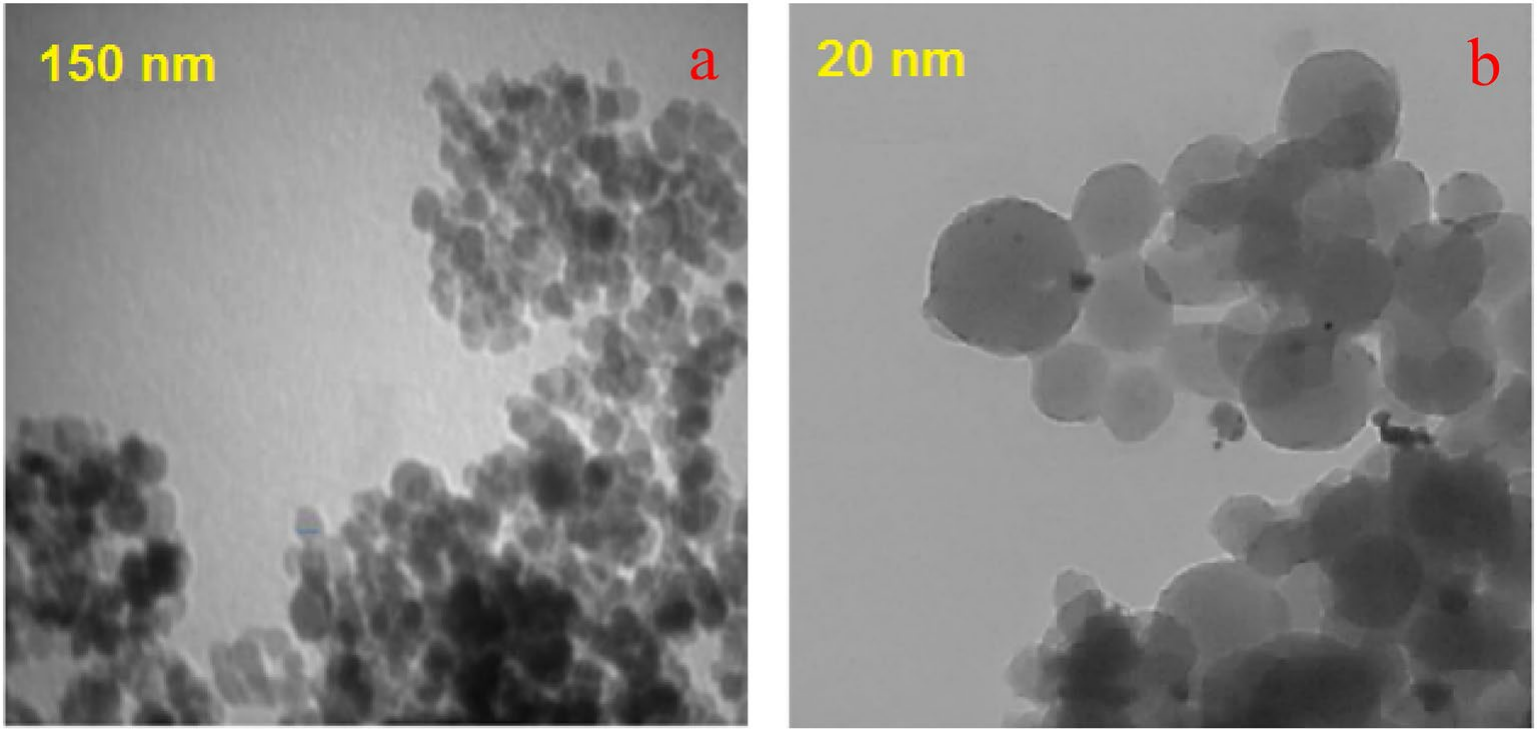
The TEM images (**a**, **b**) of Pd-DPyE@MCM-41@MNP.

As part of our continuing research effort to create novel synthetic techniques, after successfully fabricating the catalyst, we examined the surface morphology using SEM and TEM images (Figs. 1 and 2). The SEM images (Fig. 1a,b), of the synthesized Pd-DPyE@MCM-41@MNP showed that the catalyst was uniformly smaller than 60 nm in size. The majority of the particles share the same quasi-spherical form. Moreover, the particle size distribution of the Pd-DPyE@MCM-41@MNP revealed that these nanoparticles have a size in the range of 15–85 nm and a mean diameter of 53.16 nm (Fig. 1c).

The TEM images of the Pd-DPyE@MCM-41@MNP nanoparticles demonstrated the formation of Pd nanoparticles with almost spherical shape on the modified MCM-41@MNP nanoparticles surfaces (Fig. 2a,b). The little black patches in the image might be ascribed to local Pd concentrations, most likely found in the channels (Supplementary Fig. S1).

**Figure 3:**
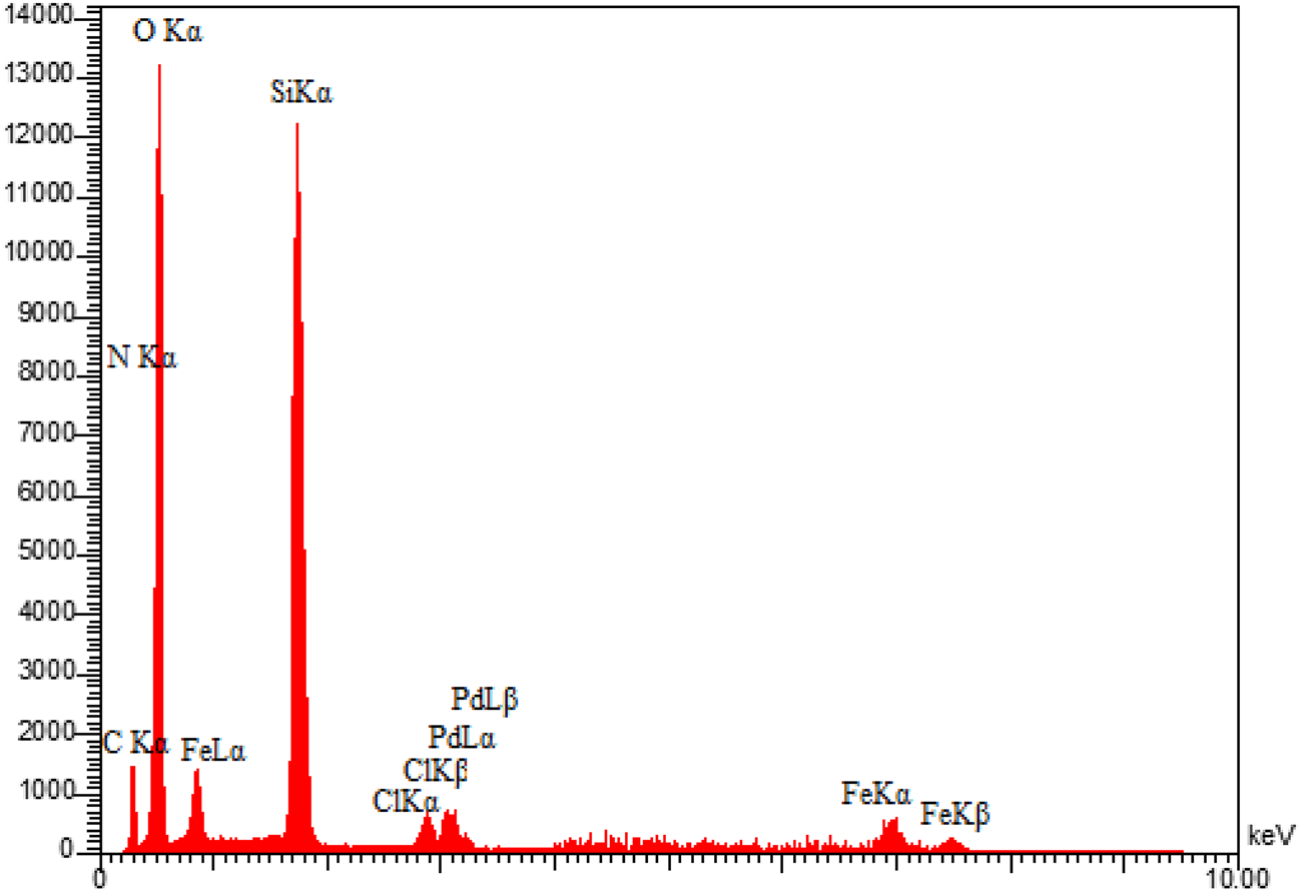
EDX pattern of Pd-DPyE@MCM-41@MNP.

**Figure 4:**
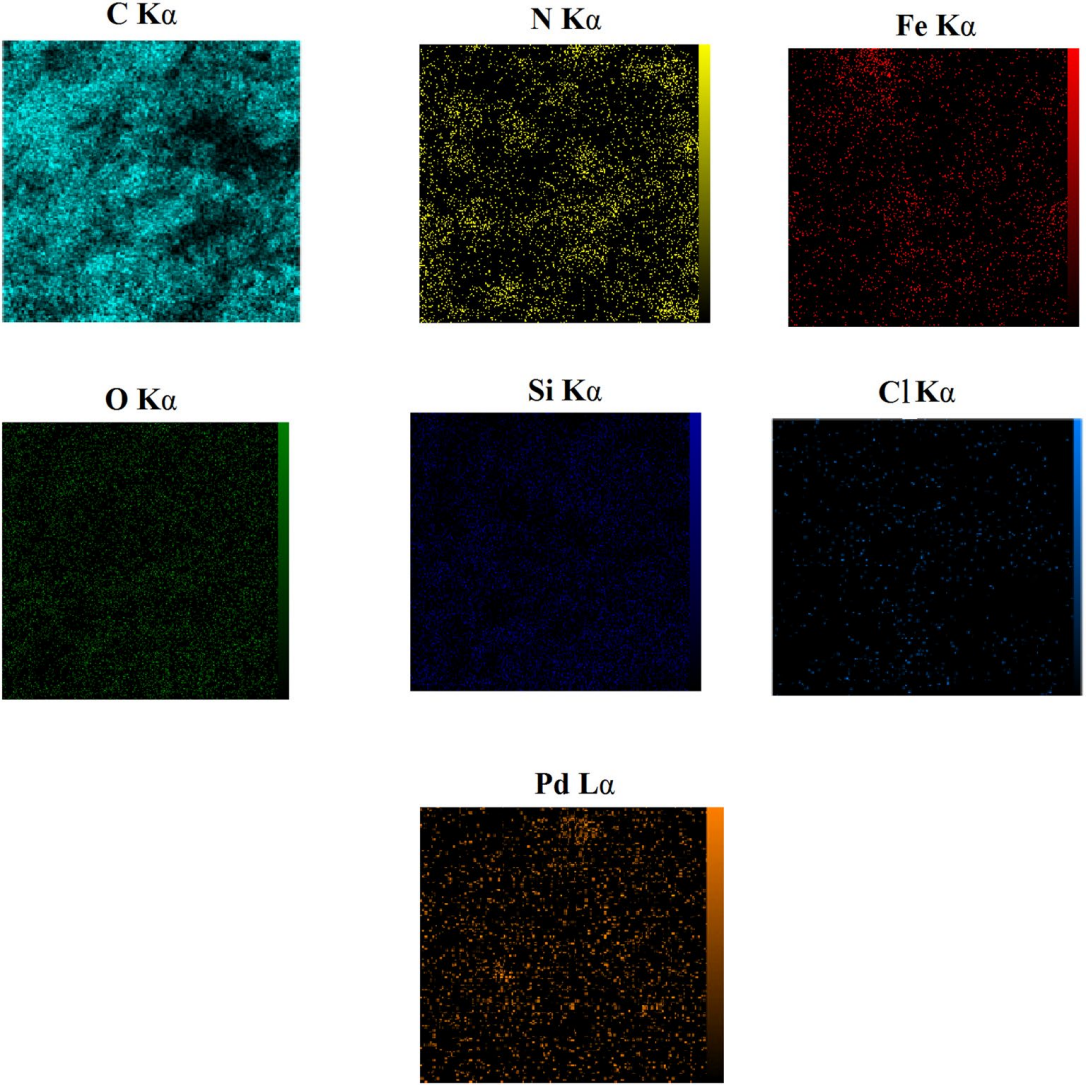
Elemental mapping images of Pd-DPyE@MCM-41@MNP.

Energy-dispersive X-ray spectroscopy (EDX) was used to analyze the presence of different elements in the produced sample. As demonstrated in Fig. 3, the presence of Pd, Fe, N, C, Si, O and Cl species in the EDX analysis of this synthesized catalyst supported the immobilization of Pd complex on MCM-41@MNP. Furthermore, the elemental mapping analysis confirms the findings of the EDS investigation. As illustrated in Fig. 4, the elemental mapping analysis revealed a homogenous distribution of all components. Also, the exact amount of Pd immobilized on DPyE@MCM-41@MNP per gram of the catalyst was achieved by inductively coupled plasma atomic emission spectroscopy (ICP-OES) and found to be 2.500 × 10^−3^ mol g^−1^.

**Figure 5:**
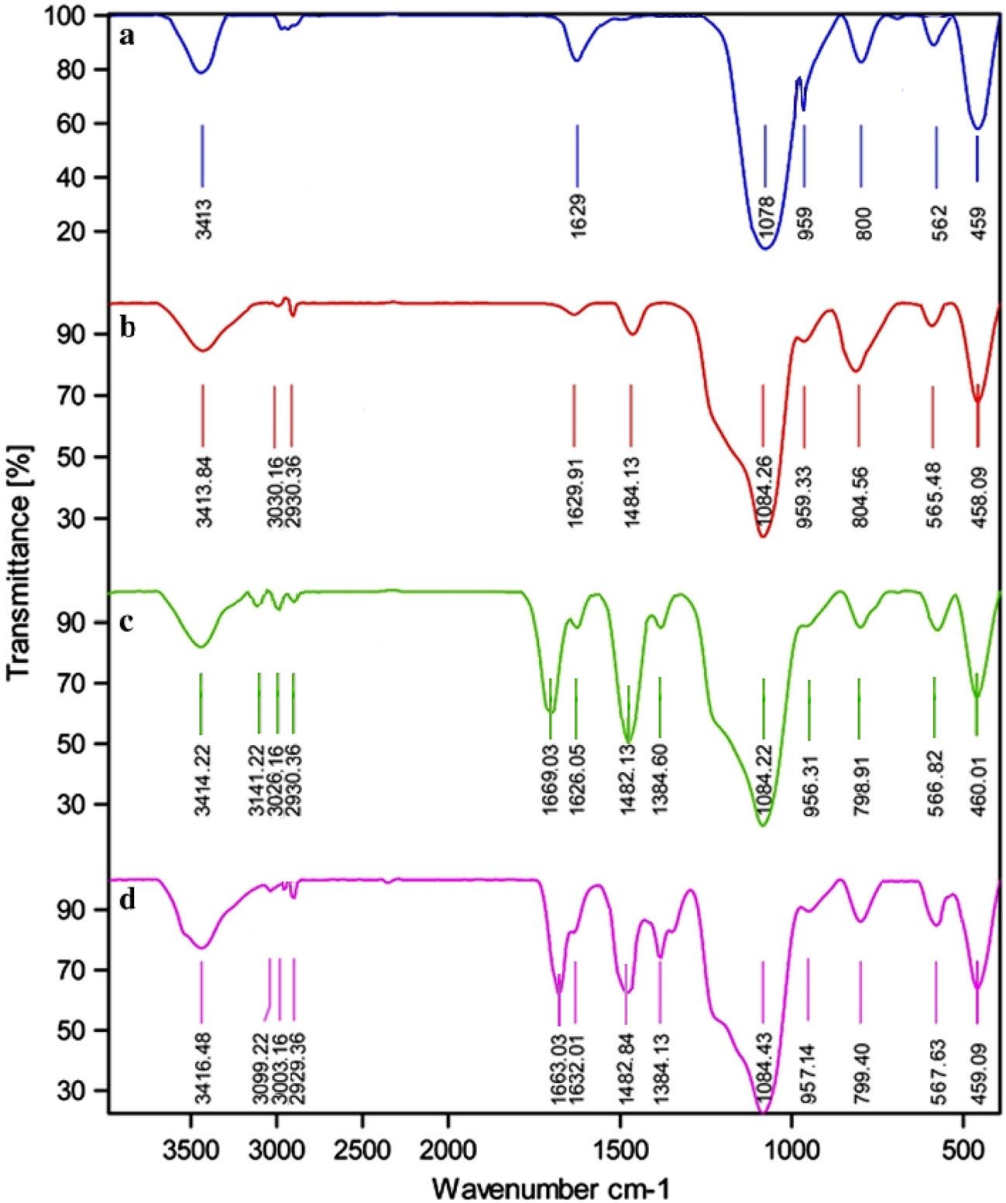
FT-IR spectra of MCM-41@MNP (**a**), nPr-Cl@MCM-41@MNP (**b**), DPyE@MCM-41@MNP (**c**), and Pd-DPyE@MCM-41@MNP (**d**).

In Fig. 5, the FT-IR spectra of MCM-41@MNP (a), nPr-Cl@MCM-41@MNP (b), DPyE@MCM-41@MNP (c), and Pd-DPyE@MCM-41@MNP (d), are shown, and the results are compiled in Table 1. According to these findings, the catalyst components are connected to each other and the desired skeleton is formed (i.e., Pd-DPyE@MCM-41@MNP). It should be noted that the palladium linking to DPyE@MCM-41@MNP was approved by the change of C=N vibration stretching in Pd-DPyE@MCM-41@MNP (1663 cm^−1^), to a lower wavenumber compared to DPyE@MCM-41@MNP (1669 cm^−1^), that is the cause for this shift is the coordination of palladium to DPyE onto functionalized MCM-41@MNP^16,61^.

**Table 1:** FT-IR data of MCM-41@MNP (a), nPr-Cl@MCM-41@MNP (b), DPyE@MCM-41@MNP (c), and Pd-DPyE@MCM-41@MNP (d).

Wavenumber (cm^−1^)	Assignments
562 (a), 565 (b), 566 (c), 567 (d)	Fe–O stretching
800 (a), 804 (b), 798 (c), 799 (d)	Si–O–Si symmetric stretching
959 (a), 959 (b), 956 (c), 957 (d)	Si–O–Fe stretching
1078 (a), 1084 (b), 1084 (c), 1084 (d)	Si–O–Si asymmetric stretching
1384 (c), 1384(d)	C–N stretching
1669 (c), 1663 (d)	C=N stretching
1482 (c), 1482 (d)	C=C stretching
2930 (b), 2930 (c), 2929 (d)	C–H symmetric stretching
3141 (c), 3100 (d)	C–H symmetric stretching =
3413 (a), 3413 (b), 3414 (c), 3416 (d)	OH stretching on the surface of the SiO_2_ and Fe_3_O_4_

**Figure 6:**
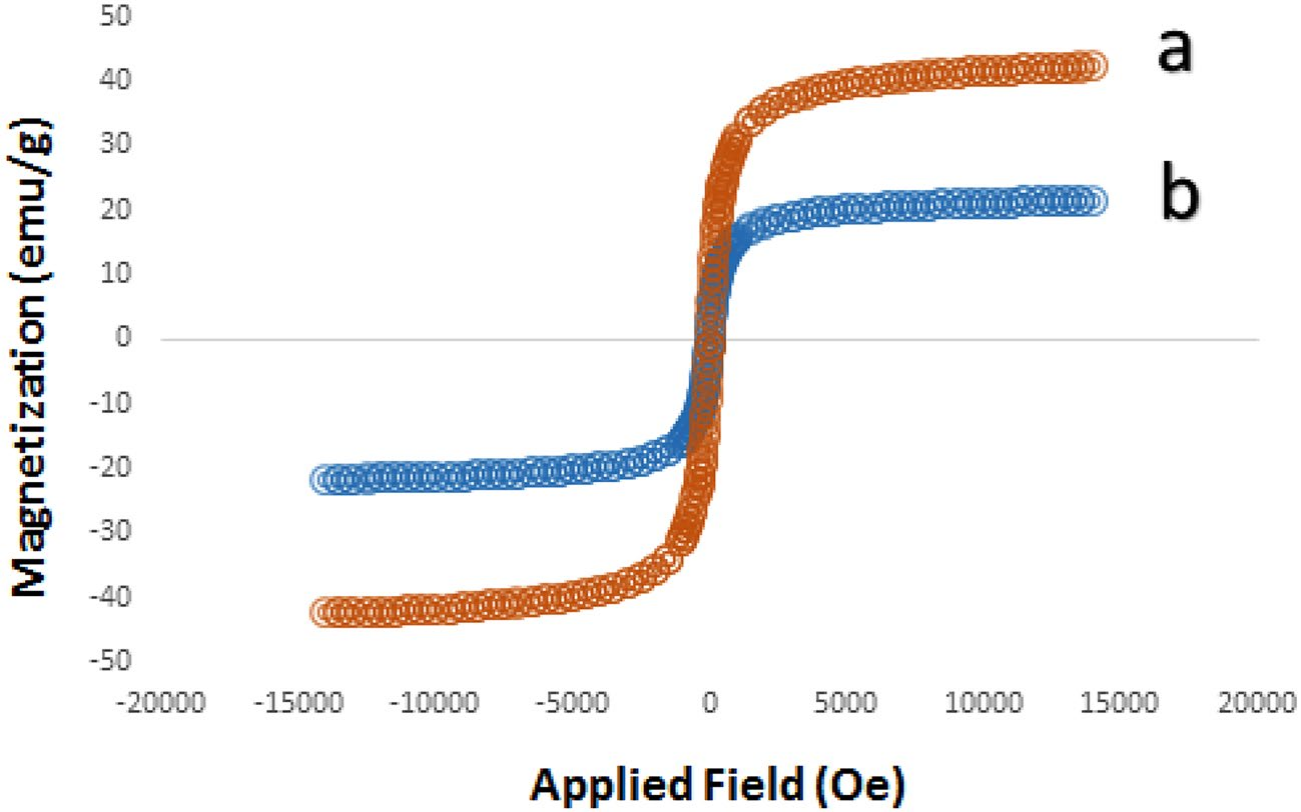
Magnetic patterns of MCM-41@MNP (**a**) and Pd-DPyE@MCM-41@MNP (**b**).

The magnetic characteristics of MCM-41@MNP and Pd-DPyE@MCM-41@MNP were studied using the VSM method. Pd-DPyE@MCM-41@MNP has a lower magnetic value than MCM-41@MNP, as seen in Fig. 6a,b. This is because organic molecules and complex palladium protect the MCM-41@MNP surface. However, its magnetic property was such that it was easily separated from the reaction mixture by magnetic decantation during the separation process.

**Figure 7:**
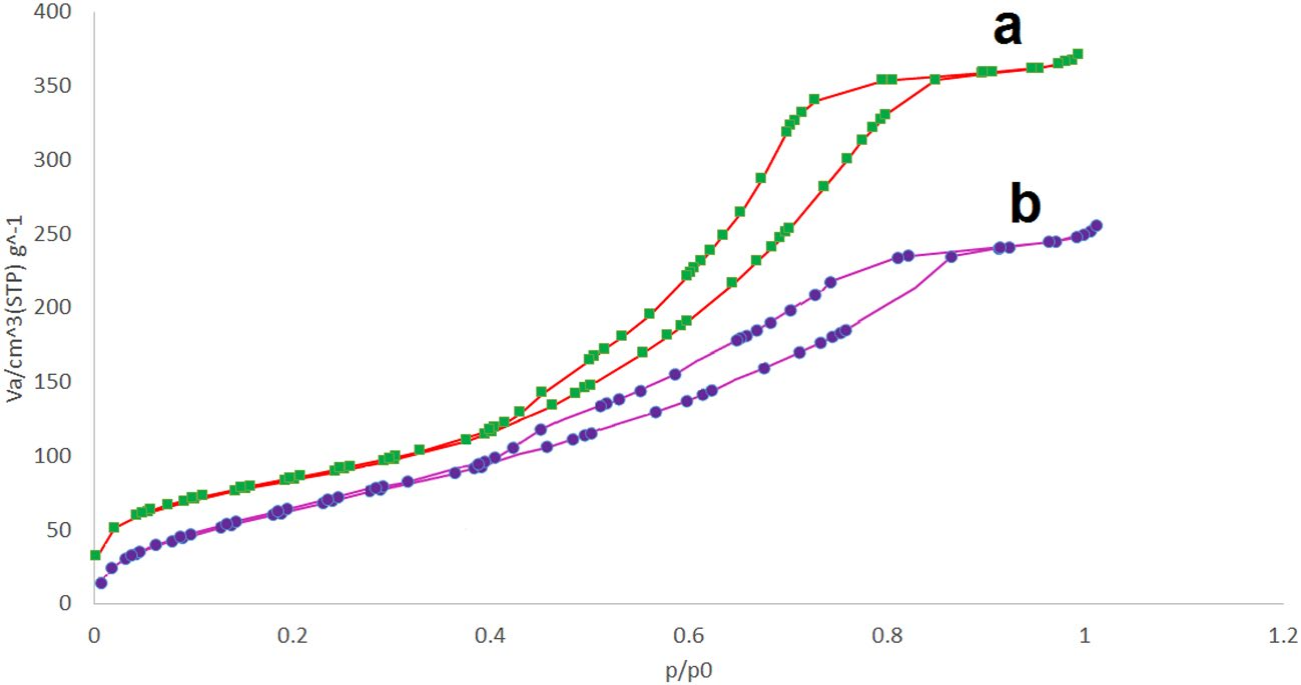
Nitrogen adsorption–desorption isotherms of MCM-41@MNP (**a**) and Pd-DPyE@MCM-41@MNP (**b**).

The nitrogen adsorption-desorptions of MCM-41@MNP and Pd-DPyE@MCM-41@MNP are depicted in Fig. 7a,b. The samples have the form of type IV curves, which are typical of mesoporous materials (according to the IUPAC classification). For MCM-41@MNP and Pd-DPyE@MCM-41@MNP, the corresponding Brunauer–Emmett–Teller (BET) surface areas are 529.8 and 367.2 m^2^/g, respectively. Pd-DPyE@MCM-41@MNP has a smaller surface area than MCM-41@MNP. This can be conveniently attributed to grafting organic materials and anchoring Pd-complex onto MCM-41@MNP mesoporous channels. Table 2 provides an overview of the samples characteristics, which agrees with the literature^62^.

**Table 2:** Textural properties of MCM-41@MNP and Pd-DPyE@MCM-41@MNP.

Sample	SBET (m^2^/g)	Pore diam by BJH method (nm)	Pore vol (cm^3^/g)
MCM-41@MNP	529.8	4.354	0.6110
Pd-DPyE@MCM-41@MNP	367.2	6.712	0.4184

**Figure 8:**
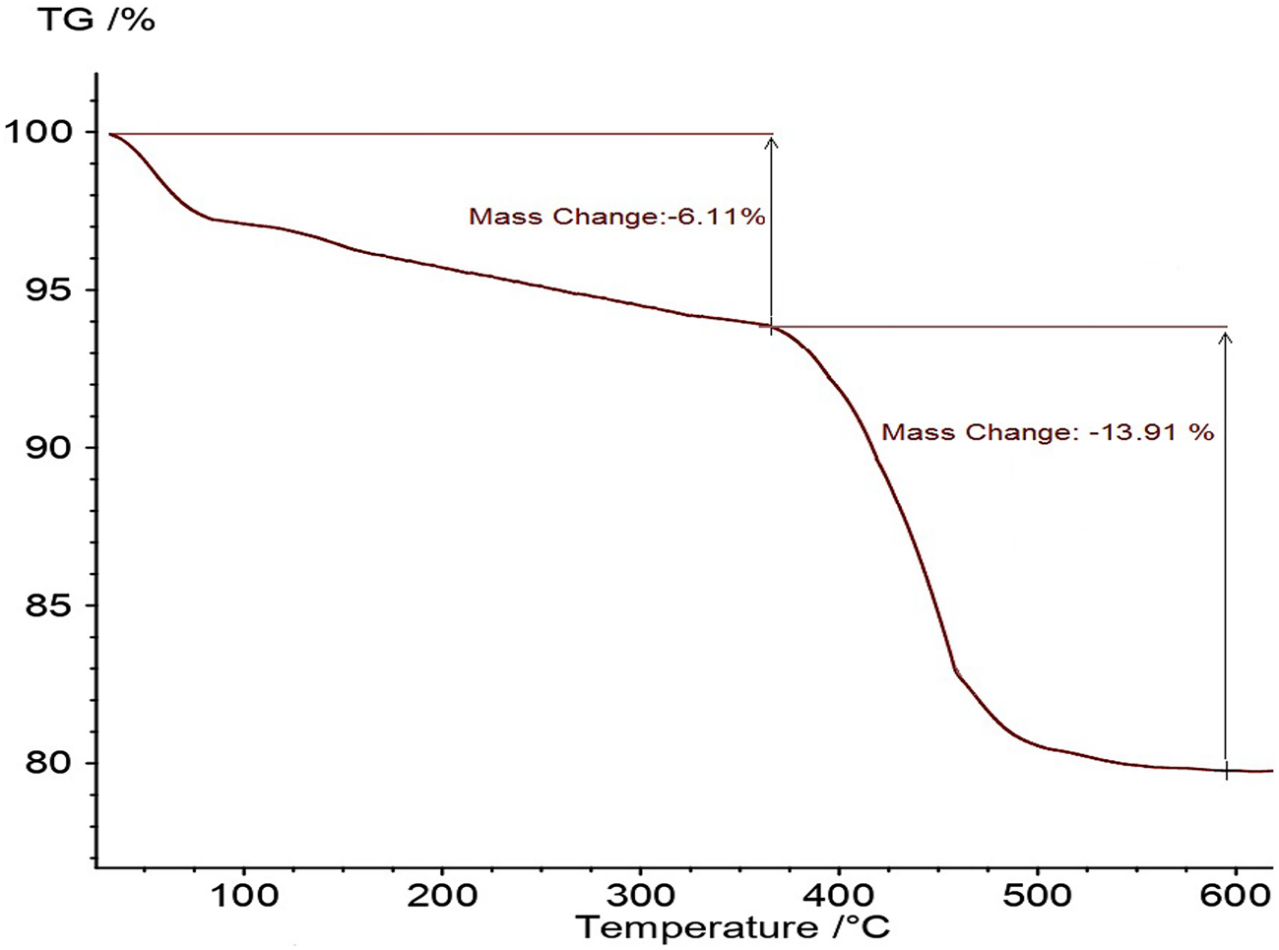
TGA analysis of Pd-DPyE@MCM-41@MNP.

Figure 8 displays the TGA diagram of Pd-DPyE@MCM-41@MNP. This curve illustrates a little weight loss in the low-temperature region (below 200 °C) of exactly 6.11%. The elimination of solvents that have been adsorbed is reflected in this weight loss. Also, the catalyst TGA diagram revealed a 13.91% weight loss in the 200–600 °C range, which is associated with the immobilized organic layers on MCM-41@MNP.

**Figure 9:**
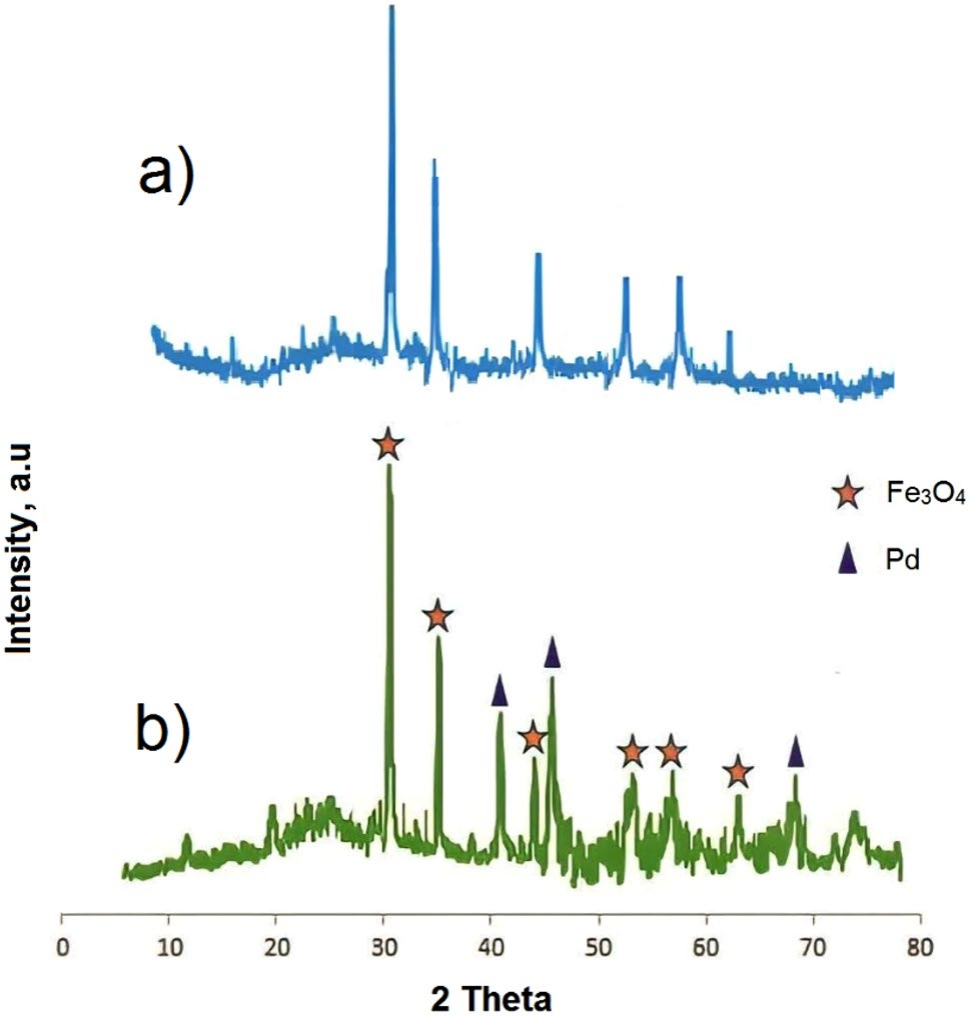
The wide-angle XRD patterns of MCM-41@MNP (**a**) and Pd-DPyE@MCM-41@MNP (**b**).

The wide-angle XRD spectra of MCM-41@MNP and Pd-DPyE@MCM-41@MNP in an extent of 2θ = 10–80° are depicted in Fig. 9a,b, respectively. In these spectra, the diffraction lines with Bragg angles at 62.72° (440), 57.01° (511), 52.88° (422), 43.22° (400), 35.23° (311), and 30.50° (220) represent the crystalline phase of iron magnetic nanoparticles^63^. The agreement of these findings with the standard pattern of MNPs indicates the fact that no change in the crystalline phase of MNPs occurred during the modification of MCM-41@MNPs. Moreover, the presence of a set of peaks with Bragg angles of 68.01° (220), 46.29° (200), and 40.09° (111) prove that Pd(0) was doped into the mesoporous channels of MCM-41@MNP (Fig. 9b)^64^. Meanwhile, the wide graph of 2θ at 20–29° degrees is correlated the silica framework in the catalyst body^65^.

**Figure 10:**
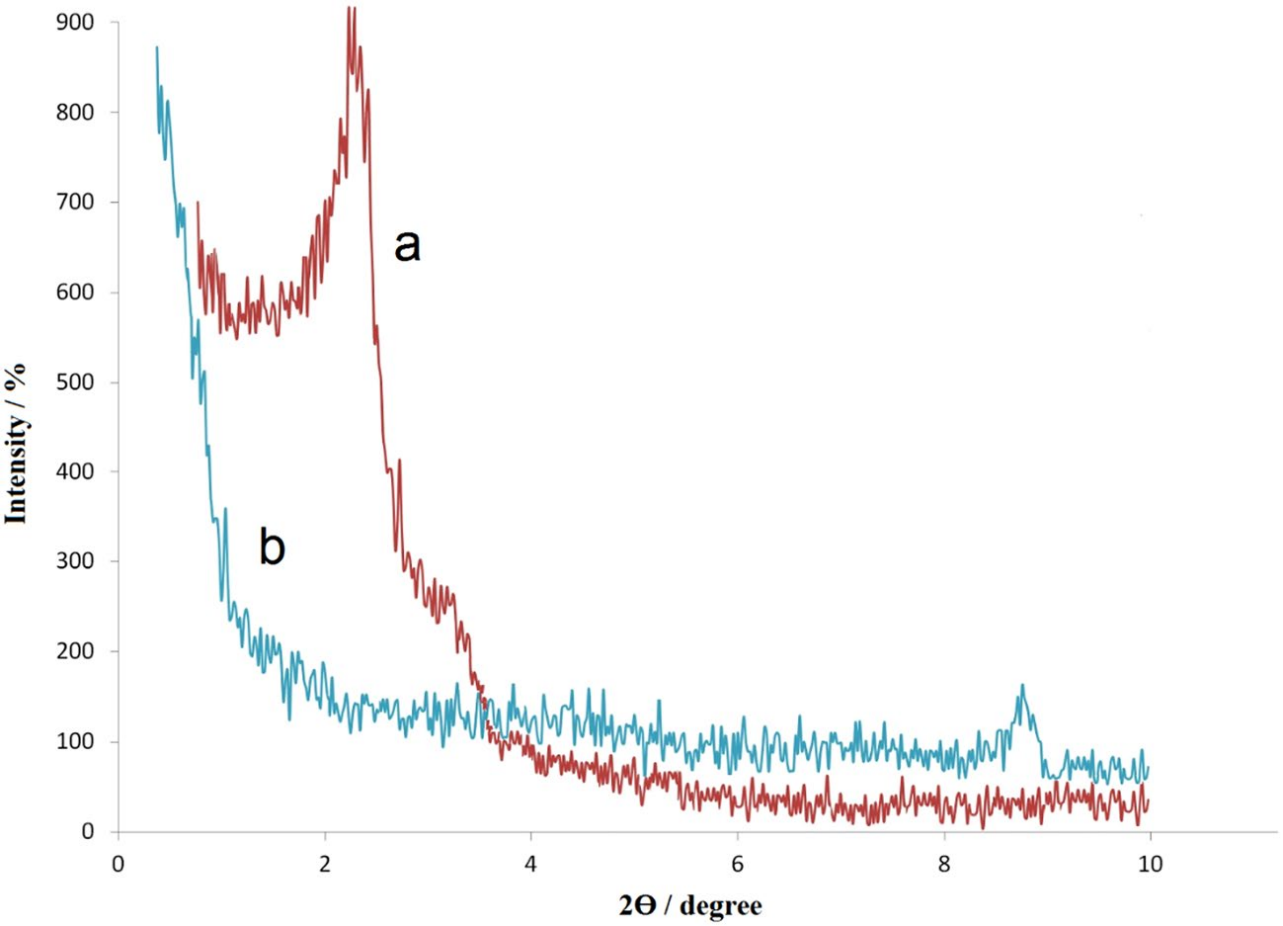
Small angle XRD patterns of MCM-41@MNP (**a**) and Pd-DPyE@MCM-41@MNP (**b**).

Figure 10 displays the small angle XRD patterns of MCM-41@MNP (a) and Pd-DPyE@MCM-41@MNP (b) to reflect the mesoporous nature of the catalyst. In this figure, the pattern of MCM-41@MNPs demonstrated a fierce peak with Bragg angles at 2θ = 2.28°, that is associated with the uniformity of mesoporous channels of MCM-41. The mentioned reflection became weaker after the creation of organic substrates and the anchoring of palladium particles on the channels ^66^.

### Evaluation of catalytic performance Pd-DPyE@MCM-41@MNP

In this section, the performance of palladium anchored organometallic catalyst on MCM-41@Fe_3_O_4_ substrate was evaluated in the synthesis of *N,N′*-alkylidene bisamide derivatives and then in the production of biphenyl derivatives through SMC reaction.

**Scheme 2:**
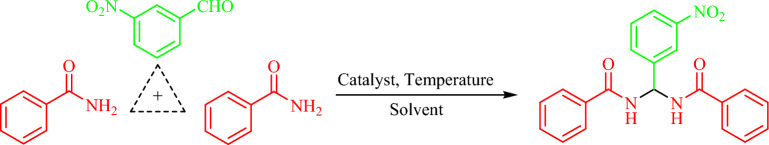
Benchmark reaction for the production of *N,N′*-alkylidene bisamides.

In the first part of this study, to evaluate the optimal conditions for the production of *N,N′*-alkylidene bisamide, the condensation of 1 equivalent 3-nitrobenzaldehyde and 2 equivalents benzamide was picked as the benchmark reaction (Scheme 2), and the impact of the nature of the solvent (using solvents e.g., H_2_O, Toluene, EtOH, THF, CH_3_CN, CHCl_3_ and EtOAc) (Table 3, entries 2–8), the type and amount of catalyst (by screening 5, 10 and 15 mg of Pd-DPyE@MCM-41@MNP, and also utilizing its components as catalyst) (Table 3, entries 11–13), and also the temperature (in the range of 25–130 °C) on its yield and time were systematically investigated. The findings were tabulated in Table 3. The comparison of the results revealed that the optimal state was obtained in solvent-free conditions using 10 mg of Pd-DPyE@MCM-41@MNP at 50 °C. It should be noted that the reaction failed under harsh circumstances in the absence of a catalyst at 130 °C (Table 3, entry 2).

**Table 3:** Evaluation of various factors in the production of *N,N′*-alkylidene bisamide derivatives.

Entry	Catalyst	Solvents (2 mL)	Temp. (°C)	Catalyst amount (mg)	Time (min)	Yield^a^ (%)
1	–		130	–	120	Trace
2	Pd-DPyE@MCM-41@MNP	H_2_O	Reflux	10	60	52
3	Pd-DPyE@MCM-41@MNP	EtOH	Reflux	10	60	70
4	Pd-DPyE@MCM-41@MNP	CH_3_CN	Reflux	10	60	80
5	Pd-DPyE@MCM-41@MNP	EtOAc	Reflux	10	60	73
6	Pd-DPyE@MCM-41@MNP	Toluene	Reflux	10	60	59
7	Pd-DPyE@MCM-41@MNP	THF	Reflux	10	60	62
8	Pd-DPyE@MCM-41@MNP	CHCl_3_	Reflux	10	60	60
9	Pd-DPyE@MCM-41@MNP	–	60	5	20	65
10	Pd-DPyE@MCM-41@MNP	–	60	10	8	98
11	Pd-DPyE@MCM-41@MNP	–	50	10	8	98
12	Pd-DPyE@MCM-41@MNP	–	50	15	8	98
13	Pd-DPyE@MCM-41@MNP	–	50	5	20	53
14	Pd-DPyE@MCM-41@MNP	–	25	10	120	42
15	di(4-pyridyl)ethylene	–	60	10	8	Trace
16	MNP	–	60	10	8	Trace
17	MCM-41@MNP	–	60	10	8	Trace
18	Pd(OAc)_2_	–	60	10	8	Trace

^a^Isolated yield.

Next, various derivatives of *N,N′-*alkylidene bisamides were fabricated via the condensation of acetamide (or benzamide) with aryl aldehydes in the best circumstances (Table 4, **1a**-**13a**). As the data in Table 4 reflected, the desired structures were synthesized in short times (8–20 min) with high efficiency (90–98%), using a variety of benzaldehyde derivatives substituted in different positions with aromatic ring activating and aromatic ring deactivating groups. The findings confirmed the wide range and effective performance of Pd-DPyE@MCM-41@MNP to catalyze the aforementioned condensation.

**Table 4:** Pd-DPyE@MCM-41@MNP-catalyzed in the production of *N*,*N′*-alkylidene bisamides.


Entry	R	Ar	Time (min)	Yield^a^ (%)	TON	TOF (min^-1^)	M.p. °C (Lit.)
**1a**	C_6_H_5_	3-O_2_NC_6_H_4_	8	98	39.200	4.900	231–233 (230–232) ^33^
**2a**	C_6_H_5_	4-O_2_NC_6_H_4_	8	98	39.200	4.900	256–258 (257–258) ^33^
**3a**	C_6_H_5_	C_6_H_5_	9	98	39.200	4.355	219–221 (220–221) ^33^
**4a**	C_6_H_5_	2,4-Cl_2_C_6_H_3_	16	94	37.600	2.350	191–193 (192–193) ^33^
**5a**	C_6_H_5_	4-ClC_6_H_4_	12	96	38.400	3.200	241–243 (241–242) ^33^
**6a**	C_6_H_5_	4-MeC_6_H_4_	14	94	37.600	2.685	239–240 (240–241) ^33^
**7a**	C_6_H_5_	2-BrC_6_H_4_	14	95	38.000	2.714	216–218 (215–217) ^35^
**8a**	CH_3_	3-O_2_NC_6_H_4_	11	98	39.200	3.563	230–232 (230–232) ^36^
**9a**	CH_3_	C_6_H_5_	13	94	37.600	2.892	234–236 (233–236) ^37^
**10a**	CH_3_	4-O_2_NC_6_H_4_	10	96	38.400	3.840	270–272 (270–272) ^36^
**11a**	CH_3_	4-MeOC_6_H_4_	20	90	36.000	1.800	215–217 (216–219) ^36^
**12a**	CH_3_	4-MeC_6_H_4_	17	92	36.800	2.164	271–273 (270–273) ^36^
**13a**	CH_3_	4-ClC_6_H_4_	14	95	38.000	2.714	254–256 (254–257) ^36^

^a^Isolated yield.

**Scheme 3:**
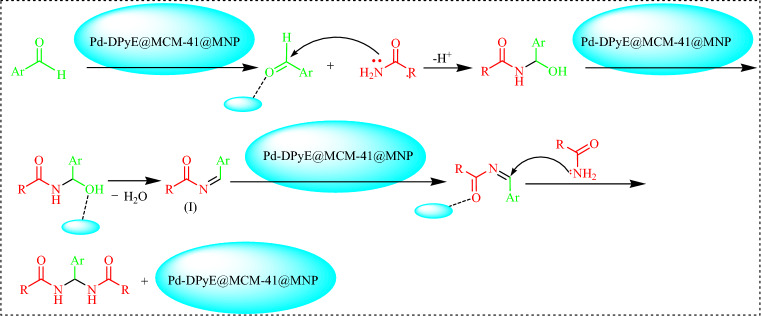
Possible mechanism of the synthesis of symmetrical *N,N′*-alkylidine bisamide.

Scheme 3 proposes a reasonable mechanism based on existing literature^36^. Pd-DPyE@MCM-41@MNP starts the process of activating aldehydes. Intermediate **I** is created by adding amides to an activated aldehyde and removing H_2_O. When a second amide molecule is added nucleophilically to activated intermediate **I**, symmetrical *N,N′-*alkylidine bisamide products are formed.

**Scheme 4:**
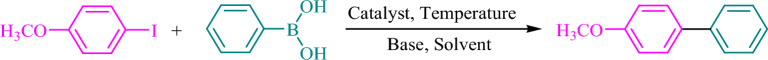
Benchmark in SMC reaction.

In the second step of this study, in order to preliminary screening of the reaction, the SMC reaction of 1 equivalent 4-methoxy iodobenzene (**4b**) with 1 equivalent benzeneboronic acid was considered as a benchmark (Scheme 4), for optimizing the reaction factors e.g., base, solvent, amount of catalyst and temperature (Table 5). Monitoring the effects of temperature in the range of 25–70 °C in various solvents (including EtOH, PEG-400, CH_3_CN, DMF and DMSO) displayed that by taking into account the criteria of green chemistry in the discussion of energy adjustment, the optimal temperature for the reaction progress was 60 °C in PEG-400 (Table 5, entry 2 vs. entries 13–15); because at a temperature of 70 °C, the efficiency of the desired derivative did not increase clearly compared to the temperature of 60 °C, and also at ambient temperature and 50 °C, the yield of the desired product dropped clearly. In the continuation of the benchmark reaction screening using various kinds bases such as Na_2_CO_3_, NaHCO_3_, DABCO and KOH (Table 5, entries 2–5), it has shown that with the help of Na_2_CO_3_, the catalyst has the highest performance and the highest yield of the coupled product is obtained. Furthermore, additional experiments proved that the amount of 6 mg of catalyst was adequate to ensure a clean and complete transformation (Table 5, entry 2). Once the amount was decreased to 3 mg, the efficiency reduced to 79% in 50 min (Table 5, entry 7). On the other hand, utilizing 9 mg of catalyst did not considerably increase the efficiency (Table 5, entry 8). These observations showed that it is necessary to use the appropriate base and temperature for this catalytic transformation because the model reaction failed in the absence of each of these two factors (Table 5, entries 1 and 6). Examining the above observations led us to consider the use of 6 mg of catalyst at 60 °C with Na_2_CO_3_ base (3 mmol) in PEG solvent (2 mL) as the optimal mode (Table 5, entry 2).

**Table 5:** Screening of various parameters in the SMC reaction (Reaction conditions: 4-methoxy iodobenzene (1 mmol), benzeneboronic acid (1 mmol), base (3 mmol), solvent (2 mL)).

Entry	Base (mmol)	Solvents (mL)	Temp. (°C)	Catalyst (mg)	Time (min)	Yield^a^ (%)
1	–	PEG	60	6	480	–
2	Na_2_CO_3_	PEG	60	6	20	97
3	NaHCO_3_	PEG	60	6	20	71
4	DABCO	PEG	60	6	20	63
5	KOH	PEG	60	6	20	69
6	Na_2_CO_3_	PEG	60	–	1440	–
7	Na_2_CO_3_	PEG	60	3	50	79
8	Na_2_CO_3_	PEG	60	9	20	98
9	Na_2_CO_3_	EtOH	60	6	20	68
10	Na_2_CO_3_	CH_3_CN	60	6	20	79
11	Na_2_CO_3_	DMF	60	6	20	64
12	Na_2_CO_3_	DMSO	60	6	20	69
13	Na_2_CO_3_	PEG	25	6	240	33
14	Na_2_CO_3_	PEG	50	6	40	78
15	Na_2_CO_3_	PEG	70	6	20	98

^a^Yield of isolated product.

By optimizing the reaction conditions, we developed the range of performance and activity of the DPyE@-MCM-41@MNP palladium-based organometallic catalyst in the SMC reaction using different (hetero)aryl bromides, iodides, and chlorides substituted with electron-releasing or electron-absorbing groups along with benzeneboronic acid (Table 6). The results presented in Table 6 demonstrate the catalyst's excellent efficiency in reactions involving aryl iodides and aryl bromides with both electron-donating and electron-accepting groups. As expected, reactions involving aryl chlorides resulted in the formation of corresponding biphenyls over a longer reaction period. However, the presence of para-substituents led to faster product formation compared to ortho-substituents, attributed to reduced steric hindrance (Table 6, Input 2b vs. 3b and entry 4b vs. 5b). Notably, the results of two reactions of 1-Bromo-4-chlorobenzene (Table 6, entry 17b), and 1-Chloro-4-iodobenzene (Table 6, entry 18b) with benzeneboronic acid, highlighted the catalyst’s selectivity in the SMC reaction with di-haloarenes; because the coupling occurred only with the bromo and iodo functional groups, and the chloro functional group remained untouched in both reactions.

**Table 6:** Preparing various derivatives in the SMC reaction via Pd-DPyE@MCM-41@MNP.


Entry	(Hetero)Aryl halide	Time (min)	Yield^a^ (%)	TON	TOF (min^−1^)	M.p. (°C)	
						Found	Reported
**1b**	Iodobenzene	10	98	65.333	6.533	68–70	68–70 ^56^
**2b**	4-Iodotoluene	15	98	65.333	4.355	47–49	46–48 ^57^
**3b**	2-Iodotoluene	25	95	63.333	2.533	Light yellow liquid	Light yellow liquid ^57^
**4b**	4-Iodoanisole	20	97	64.666	2.233	86–88	87–89 ^57^
**5b**	2-Iodoanisole	30	96	64.000	2.133	Colorless oil	Colorless oil ^57^
**6b**	2-Iodobenzoic acid	25	96	64.000	2.560	110–112	111–113 ^57^
**7b**	Bromobenzene	15	97	64.666	4.311	67–69	68–70 ^56^
**8b**	4-Bromotoluene	25	96	64.000	2.560	47–49	46–48 ^57^
**9b**	4-Bromoanisole	25	95	63.333	2.533	86–88	87–89 ^57^
**10b**	4-Bromobenzonitrile	30	97	64.666	2.155	84–86	83–85 ^57^
**11b**	4‐Bromonitrobenzene	20	98	65.333	3.266	111–113	112–114 ^56^
**12b**	4-Bromothiophenol	40	93	62.000	1.550	111–112	110–111 ^58^
**13b**	4-Bromobenzoic acid	20	95	63.333	3.166	226–228	225–227 ^59^
**14b**	4-Bromoacetophenone	25	94	62.666	2.506	115–117	116–118 ^56^
**15b**	4-Bromoaniline	30	96	64.000	2.133	50–52	49–51^57^
**16b**	3‐Bromobenzaldehyde	50	91	60.666	1.213	52–54	52–54 ^57^
**17b**	1-Bromo-4-chlorobenzene	60	90	60.000	1.000	78–80	77–79 ^57^
**18b**	1-Chloro-4-iodobenzene	30	94	62.666	2.088	78–80	77–79 ^57^
**19b**	Chlorobenzene	50	90	60.000	1.200	78–70	69–70 ^57^
**20b**	2,4-Dinitrochlorobenzene	90	89	59.333	0.659	76–78	75–78 ^57^
**21b**	2-Iodopyridine	20	92	61.333	3.066	Light oil	Light oil ^67^
**22b**	3-Iodopyridine	20	94	62.666	3.133	Light oil	Light oil ^68^
**23b**	4-Iodopyridine	15	97	64.666	4.311	74–76	75–77 ^69^
**24b**	3-Amino-6-bromopyridine	25	91	60.066	2.426	100–102	102.2–104.4 ^70^
**25b**	7-Chloro-4-iodoquinoline	35	92	61.333	1.752	71–73	73–74 ^71^
**26b**	2,4-Dichloropyrimidines	140	86	57.333	0.409	83–85	84–86 ^72^

^a^Isolated yield.

**Scheme 5:**
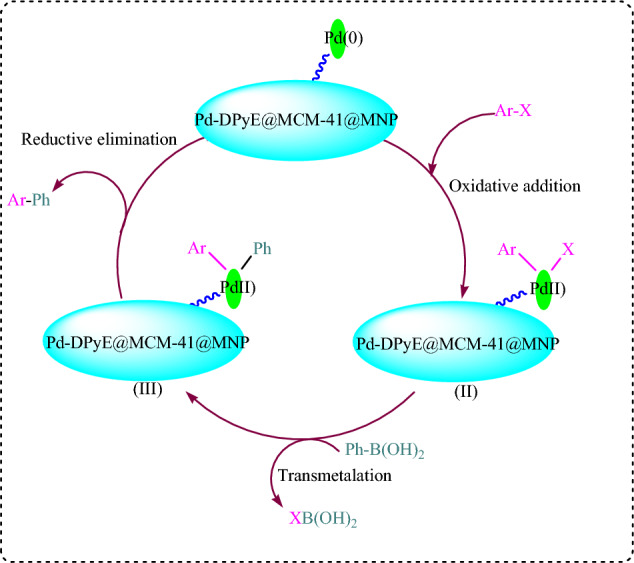
Possible mechanism of the SMC reaction.

Scheme 5 illustrates a possible mechanism for the Pd-DPyE@MCM-41@MNP SMC reaction. The first stage in the previously described procedure is the oxidative addition of copper to the aryl halide, resulting in the formation of the organocopper intermediate (**II**). Intermediate (**III**) is produced via transmetallation of (**II**). The catalytic cycle could proceed because the intermediate (**III**) was eliminated reductively, which led to the synthesis of the product and the regeneration of the catalyst ^2^.

### Reusability of catalyst

**Figure 11:**
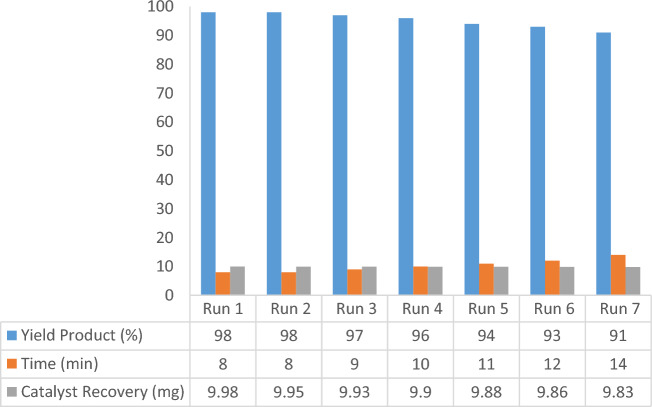
Recyclability of Pd-DPyE@MCM-41@MNP in the synthesis of compound **1a**.

**Figure 12:**
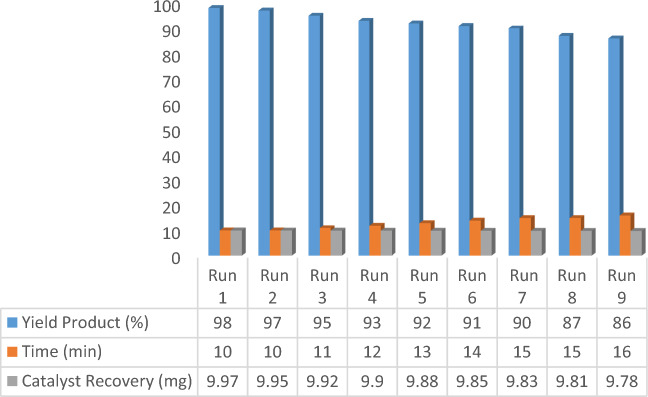
Recyclability of Pd-DPyE@MCM-41@MNP in the synthesis of compound **1b**.

The ability of magnetic nanoparticles to be recovered and reused several times is one of its most significant features. In this regard, the catalyst was separated from the reaction mixture using an external magnet after the reaction was finished, it washed with EtOH and EtOAc. After drying at 60 °C, ready for the subsequent run. After recovery, we investigated the activity of Pd-DPyE@MCM-41@MNP during the synthesis of compounds (**1a**) and (**1b**). According to the findings, this magnetic nanocatalyst maintained its catalytic activity for at least seven runs when it was recovered and reused (Figs. 11 and 12).

**Figure 13:**
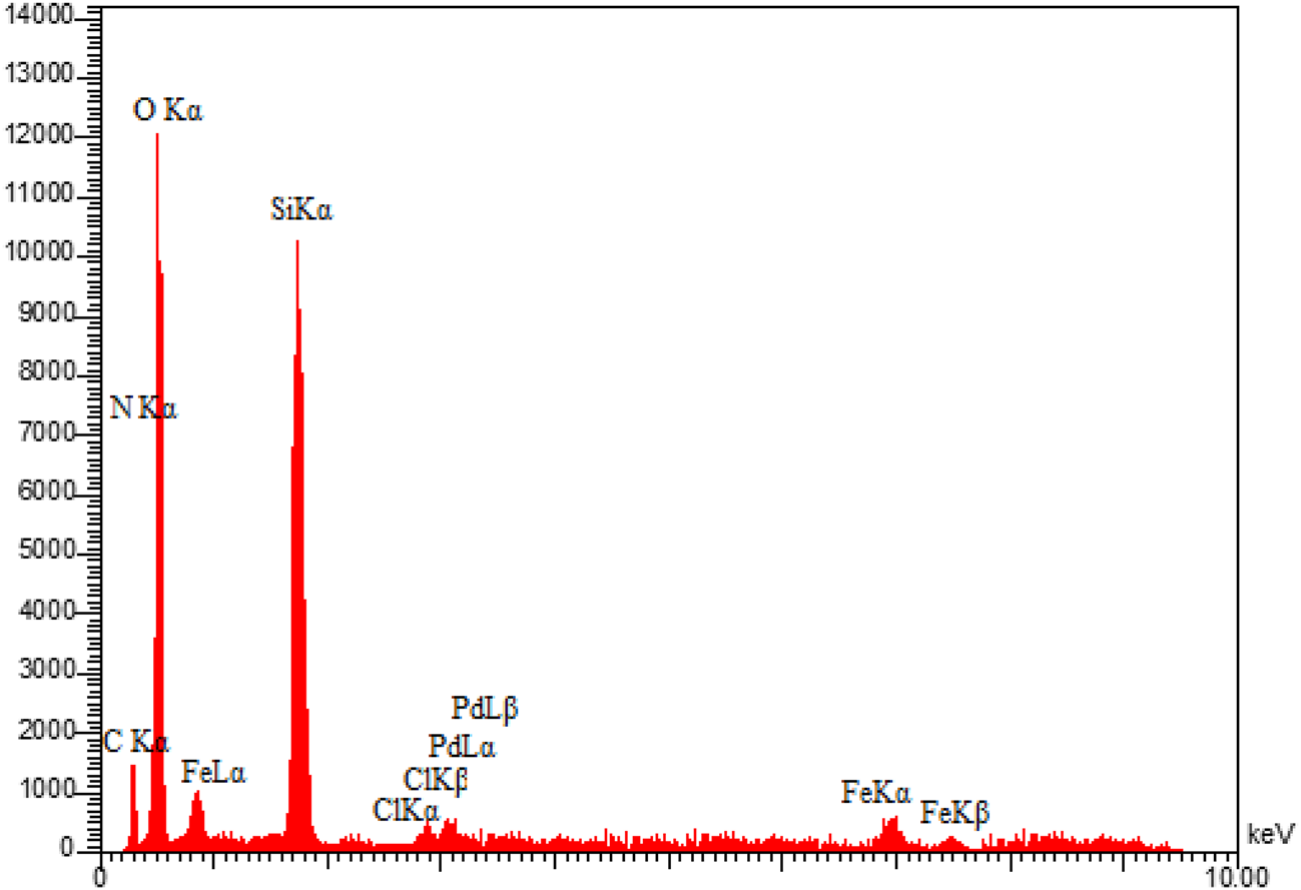
EDX pattern of consumed Pd-DPyE@MCM-41@MNP in the synthesis of compound **1a**.

**Figure 14:**
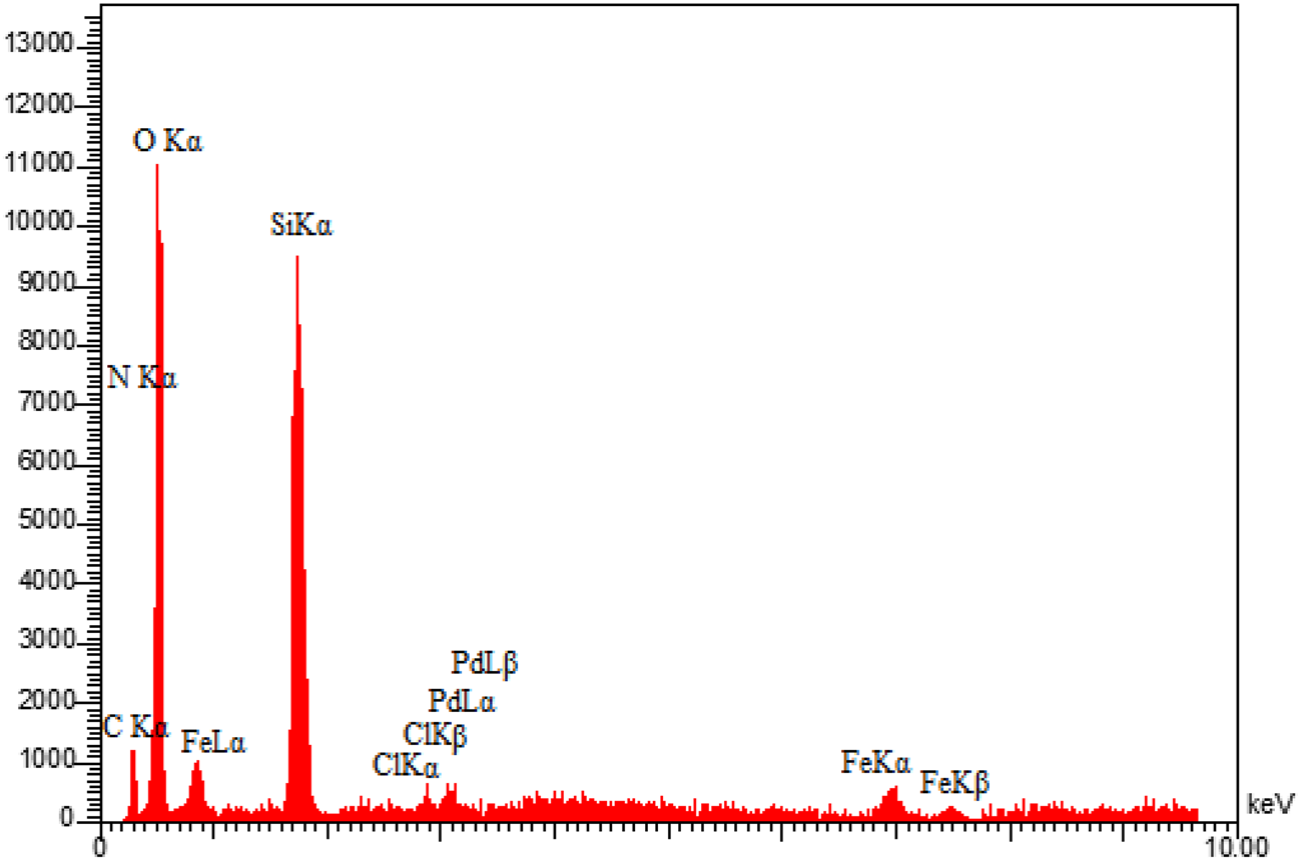
EDX pattern of consumed Pd-DPyE@MCM-41@MNP in the synthesis of compound **1b**.

**Figure 15:**
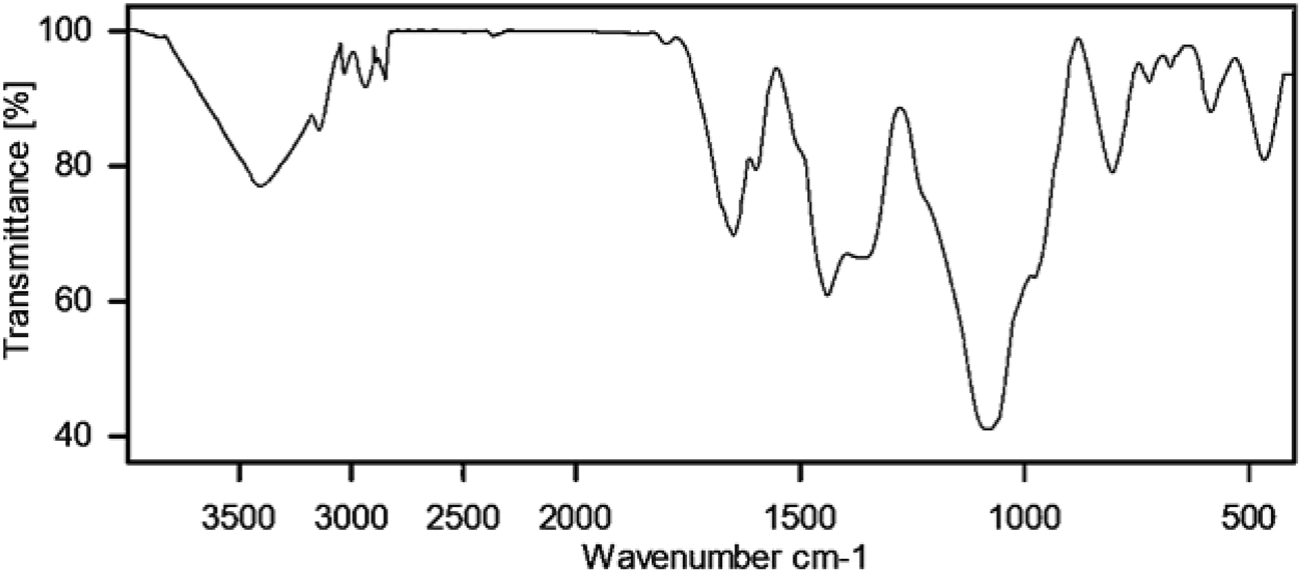
FT-IR pattern of consumed Pd-DPyE@MCM-41@MNP in the synthesis of compound **1a**.

**Figure 16:**
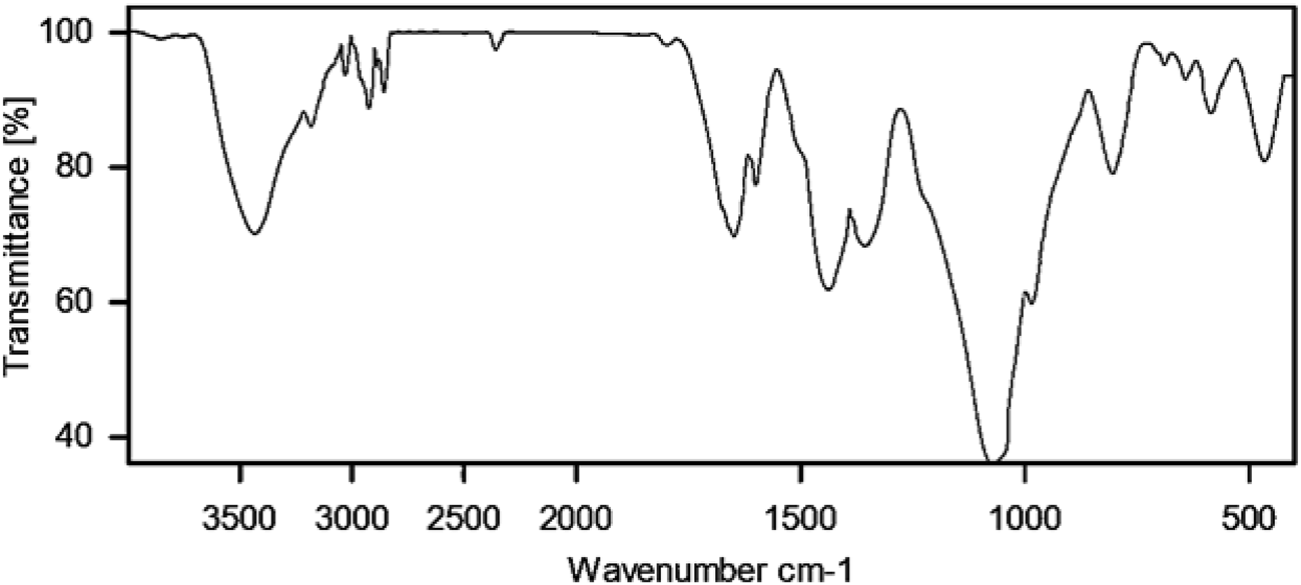
FT-IR pattern of consumed Pd-DPyE@MCM-41@MNP in the synthesis of compound **1b**.

**Figure 17:**
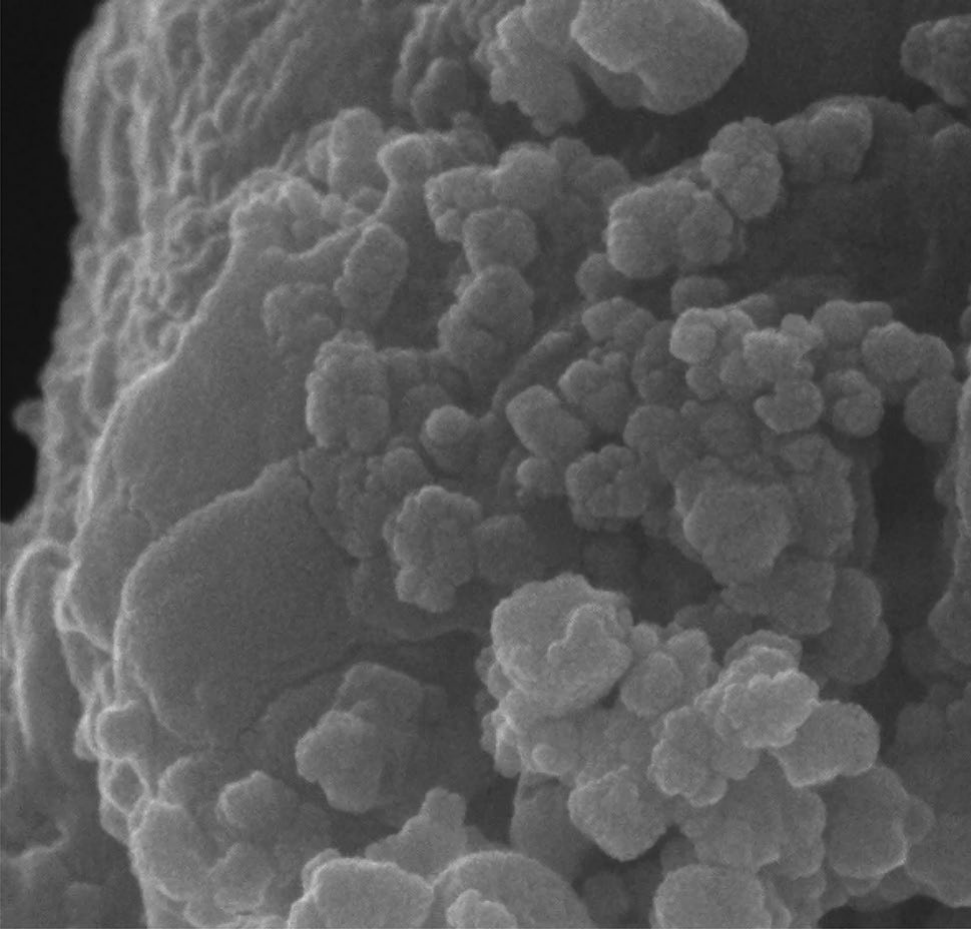
SEM micrograph of consumed Pd-DPyE@MCM-41@MNP in the synthesis of compound **1a**.

**Figure 18:**
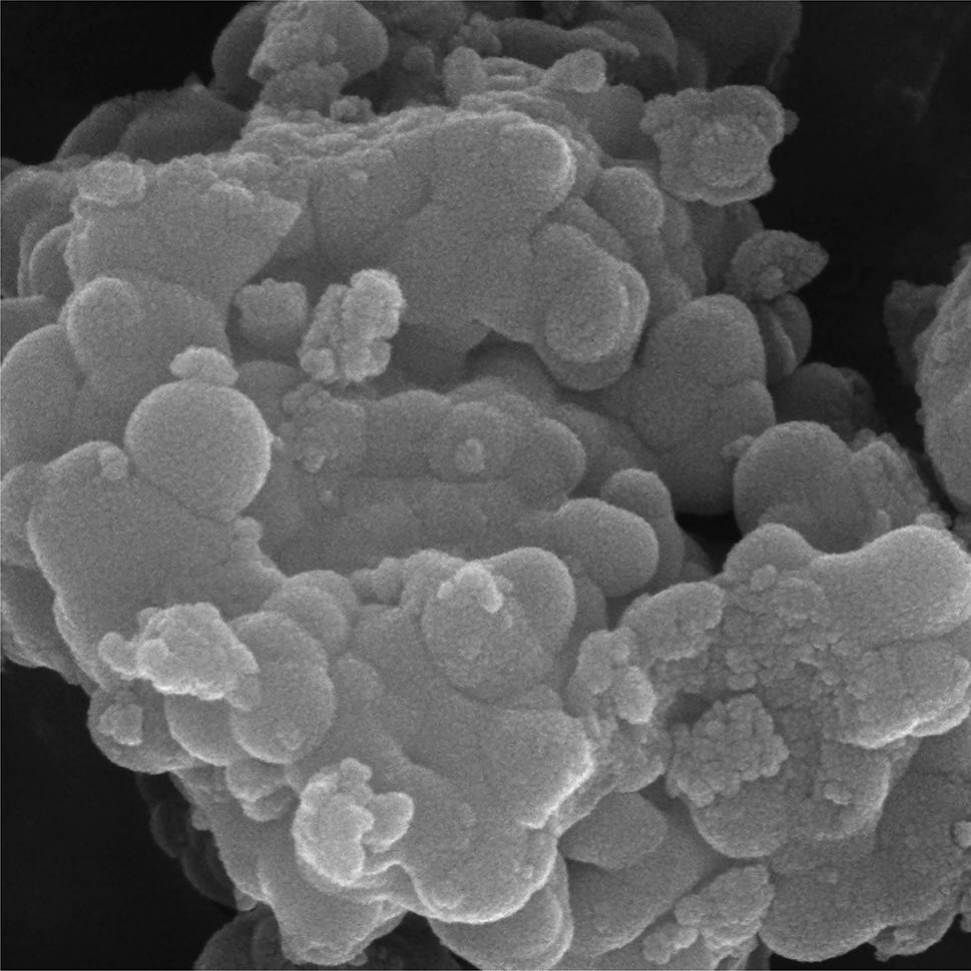
SEM micrograph of consumed Pd-DPyE@MCM-41@MNP in the synthesis of compound **1b**.

Next, after the seventh recycling in production **1a** and the ninth recycling in production **1b**, the structure of the catalyst was evaluated separately by EDX (Figs. 13 and 14), FT-IR (Figs. 15 and 16) and SEM (Figs. 17 and 18) techniques. In both ways of recycling, the matching of the findings of these analyses with the corresponding spectra of the fresh catalyst sample showed that the structure and texture of the catalyst did not change significantly during repeated use, even in the last cycle. These results support the high reproducibility of our catalyst; however, the natural wastage (0.02–0.03%) of the catalyst during recycling and the aggregation of its particles has caused a slight decrease in its performance.

### Leaching study of catalyst

Palladium washing from Pd-DPyE@MCM-41@MNP was examined through ICP-OES and a hot filtration procedure. Palladium concentrations of the fresh and reused catalysts were found to be 2.500 × 10^−3^ mol g^−1^ and 2.479 × 10^−3^ mol g^−1^, respectively, according to the ICP-OES analysis. These findings imply that this catalyst has minimal palladium leaching.

To address the variable nature of Pd-DPyE@MCM-41@MNP in the Suzuki–Miyaura coupling reaction, the hot filtration experiment was carried out in the coupling of iodobenzene and benzeneboronic acid. In this investigation, 53% of the product was produced in half the reaction time (5 min). After five minutes, the catalyst was separated and the reaction was resumed. After that, the catalyst-free reaction was carried out for a further five minutes by the filtrated solution. Consequently, only 56% of 1,1'-biphenyl was generated. These results reveal that there is no visible rise in product concentration that indicates a heterogeneous mechanism throughout the recycling process.

### Comparison

The efficiency of Pd-DPyE@MCM-41@MNP was studied by comparing our results with the previously described techniques in the synthesis of **3a** and **1b** compounds. As indicated in Table 7, Pd-DPyE@MCM-41@MNP has good results than other catalysts. Furthermore, Pd-DPyE@MCM-41@MNP has various benefits, including stability, short reaction time, high yield, and ease of separation.

**Table 7:** Comparison results of Pd-DPyE@MCM-41@MNP with other catalysts in the synthesis of **3a** and **1b** products.

Catalyst	Conditions	Type of reaction	Time (min)	Yield^a^ (%)	Ref
Pd-DPyE@MCM-41@MNP	Solvent-free, 50 °C	I	9	98	This work
Pd-DPyE@MCM-41@MNP	Na_2_CO_3_, PEG, 60 °C	II	10	98	This work
Hydroxyapatite	CH_3_CN, Reflux	I	180	92	^31^
Pd/Au NPs	K_2_CO_3_, EtOH/H_2_O, 80 °C	II	1440	88	^52^
Nano-SnCl_4_.SiO_2_	n-hexane, Reflux	I	126	90	^32^
Pd(II)-NHC complex	Cs_2_CO_3_, DMF, 100 °C	II	1440	99	^53^
ZnO/KIT-6@NiFe_2_O_4_	Solvent-free, 60 °C	I	10	90	^33^
ZrFe_2_O_4_@SiO_2_@Ade-Pd	Et_3_N, EtOH, 60 °C	II	20	99	^2^
KIT-6@NiFe_2_O_4_	Solvent-free, 60 °C	I	10	36	^33^
Pd-AcAc-Am-Fe_3_O_4_@SiO_2_	K_2_CO_3_, DMF: H_2_O, 80 °C	II	60	96	^54^
NiFe_2_O	Solvent-free, 125 °C	I	10	57	^33^
GO/Fe_3_O_4_/PAMPS/Pd	PEG-400, 80 °C	II	120	100	^55^
Polymer supported Bronsted acid ionic liquid	Toluene, Reflux	I	30	85	^34^

^a^Isolated yield.

## Conclusions

Briefly, Pd-DPyE@MCM-41@MNP as a retrievable, reusable heterogeneous organometallic nanocatalyst was prepared by immobilizing the Pd-DPyE complex on MCM-41@MNP support and characterized by a wide range of physicochemical parameters. Then, the catalytic power of Pd-DPyE@MCM-41@MNP in the preparing *N,N′*-alkylidene bisamides and SMC derivatives was assessed separately through (i) reaction of aldehydes (1 equivalent) with primary amides (2 equivalents) and, (ii) reaction between (hetero)aryl halides (1 equivalent) with benzeneboronic acid (1 equivalent) under benign and mild conditions. The findings reflected that this catalyst in both production routes successfully isolated the respective derivatives in short reaction times (8–20 min and 10–140 min, respectively) with excellent yields (90–98% and 86–98%, respectively). In addition, in routes (i) and (ii) catalysts were used in 7 and 9 consecutive runs, respectively, with an average yields of 95.28% to 92.11%, and the durability of the nanostructured skeleton was confirmed after recycling experiments with SEM and EDX techniques. Pd-DPyE@MCM-41@MNP has a high potential for catalytic conversion due to its simplicity of isolation, low cost, high efficiency, and high reproducibility/stability; consequently, its catalytic performance can be investigated in future research.

### Supplementary Information

Supplementary Information.

## Data Availability

The data that support the findings of this study are available on request from the corresponding author.
