# Supplementary material for: Synthesis of N,N′-alkylidene bisamides and Suzuki–Miyaura coupling reaction derivatives with Pd organometallic catalyst anchored to channels of mesoporous silica MCM-41
Source: Sci Rep. 2024 Apr 2;14:7688. doi: 10.1038/s41598-024-58310-5 (PMC10985085; doi:10.1038/s41598-024-58310-5)
Supplement: Supplementary file 1 — Supplementary Information. [file 41598_2024_58310_MOESM1_ESM.docx]

**Supplementary Information**

**Synthesis of *N,N'*-alkylidene bisamides and Suzuki-Miyaura coupling reaction derivatives with Pd organometallic catalyst anchored to channels of mesoporous silica MCM-41**

Sulieman Ibraheem Shelash Al-Hawary­^1­^,­ Raed Obaid Saleh^2^, Ahmed Rafiq AlBajalan^3^­,­ Normurot Fayzullaev­^4^­, Mohammed Alshuhri^5^, Saad Hayif Jasim Ali­­^6^, Ahmed Alawadi­^7,8,9^, Mohammed Abed Jawad­­^10^, Salim B. Alsaadi­^11^, Maryam Sadat Ghorayshi Nejad­­^12*^

^1^­ *Professor of Business Management, Department of Business Administration, Business School, Al al-Bayt University, P.O.BOX 130040, Mafraq, 25113, Jordan, E-mail: dr_sliman73@aabu.edu.jo; dr_sliman@yahoo.com*

^2^­ *Department of Medical Laboratory Techniques, Al-Maarif University College, Al-Anbar, Iraq, E-mail: Dr.raed.obaid@uoa.edu.iq*

^3^ *Petroleum Technology Department, Erbil Polytechnic University, Erbil­, Iraq, E-mail:* *ahmed.al_bajalan@epu.edu.iq*

^4^ *Department of Polymer Chemistry and Chemical Technology, Samarkand State University, Samarkand, 140101, Uzbekistan, E-mail: nfayzullaev1972@gmail.com*

^5^­ *Radiology and Medical Imaging Department, College of Applied Medical Sciences, Prince Sattam bin Abdulaziz University, Kharj, 11942, Saudi Arabia, E-mail: m.alshuhri@psau.edu.sa*

^6^ *Department of Medical Laboratory, College of Health and Medical Technololgy, Al-Ayen University, Thi-Qar, Iraq, E-mail*: [saad.albadry@alayen.edu.iq](mailto:saad.albadry@alayen.edu.iq)

^7^ *College of Technical Engineering, the Islamic University, Najaf, Iraq*, *E-mail:* *ahmedalawadi85@gmail.com*

^8^ *College of Technical Engineering, the Islamic University of Al Diwaniyah, Al Diwaniyah, Iraq*

*^9^ College of Technical Engineering, the Islamic University of Babylon, Babylon, Iraq*

^10^ *Department of Pharmaceutics, Al-Nisour University College, Baghdad, Iraq, E-mail: mohammed.a.medical.lab@nuc.edu.iq*

^11^ *Department of Pharmaceutics, Al-Hadi University College, Baghdad, 10011, Iraq, E-mail: salima.b@huc.edu.iq*

^12^ *Takin Shimi Sepanta Industries Co, Sirvan Industrial Zone, PO 6958140120, Ilam, Iran, E-mail: ghoreishinezhad2023@gmail.com;* [*mghoreishi1396@gmail.com*](mailto:mghoreishi1396@gmail.com)

*­Corresponding author. Maryam Sadat Ghorayshi Nejad: ­ ghoreishinezhad2023@gmail.com; [mghoreishi1396@gmail.com](mailto:mghoreishi1396@gmail.com)

**Experimental**

**Materials and devices**

All chemicals were bought from Fluka and Merck Chemical Companies. Through comparison the known compounds' melting points and NMR data with those that have been reported in the literature, the structures of the known compounds were characterized. Thin layer chromatography (TLC) was used to check the progress of the reaction. ^1^H-NMR and ^13^C-NMR spectra were recorded at frequencies of 500 and 125 MHz, respectively, using a Bruker Avance DPX FT-NMR spectrometer. The melting points of the materials in the open capillary tubes were measured with a Buchi B-545 device. Panalytical X’Pert PRO MPD device was employed to determine XRD patterns with Cu Kα radiation (λ=1.5408 Å). Devices model TESCAN MIRA3 LMH and Philips CM200 were applied for recording FE-SEM and TEM images of the nanocatalyst. A Vibrating Sample Magnetometer (VSM, ADE-DMS 1660, MA, US A) was utilized to evaluate the magnetic nature of the nanocatalyst. Nitrogen adsorption-desorption isotherms were recorded on a BELSORP-mini II surface area analyzer. A Varian ICP-OES (inductively coupled plasma atomic emission) spectrometer (model: VISTA-MPX, made in Australia) was utilized to measure the amount of Pd in the catalytic system. Thermo device (Perkin-Elmer BX-II model) (KBr plates) was employed to record FT-IR spectra. Thermogravimetric analysis (TGA) was done by Perkin Elmerpyris apparatus, with temperature rise rate of 10 °C.min^-1^ at 25-600 °C in N_2_ atmosphere.

**Selected spectroscopic data of *N,N′*-alkylidene bisamides**

**Product 2a:** *N,N′*-(4-Nitrophenylmethylene)dibenzamide;^1^H NMR (500 MHZ, DMSO-*d_6_*) δ (ppm): 9.29 (d, J = 5.5 Hz, 2H, 2NH), 8.25 (d, J = 8.2 Hz, 2H, H_Ar_), 7.96 (d, J = 7.4 Hz, 4H, H_Ar_), 7.76 (d, J = 8.4 Hz, 2H, H_Ar_), 7.56 (t, J = 6.8 Hz, 2H, H_Ar_), 7.48 (t, J = 6.9 Hz, 4H, H_Ar_),

7.13 (t, J = 7.3 Hz, 1H, CH) (Fig. S1); ^13^C NMR (125 MHZ, DMSO-*d_6_*) δ (ppm): 166.38, 148.11, 147.50, 134.01, 132.19, 128.79, 128.51, 128.08, 123.97, 59.02 (Fig. S2).

**Product 3a:** *N,N*′-(Phenylmethylene)dibenzamide;^1^H NMR (500 MHZ, DMSO-*d_6_*) *δ* (ppm): 9.05 (d, *J* = 7.7 Hz, 2H, 2NH), 7.93 (d, *J* = 7.4 Hz, 4H, H_Ar_), 7.56 (t, *J* = 7.2 Hz, 2H, H_Ar_), 7.50-7.47 (m, 6H, H_Ar_), 7.39 (t, *J* = 7.6 Hz, 2H, H_Ar_), 7.32 (t, *J* = 7.2 Hz, 1H, H_Ar_), 7.07 (t, *J* = 7.7 Hz, 1H, CH) (Fig. S3); ^13^C NMR (125 MHZ, DMSO-*d_6_*) *δ* (ppm): 166.07, 140.77, 134.32, 132.05, 128.79, 128.14, 127.97, 126.97, 59.18 (Fig. S4).

**Product 5a:** *N,N*′-(­4-Chlorophenylmethylene)dibenzamide;^1^H NMR (500 MHZ, DMSO-*d_6_*) *δ* (ppm): 9.06 (d, *J* = 8.0 Hz, 2H, 2NH), 7.92 (d, *J* = 7.8 Hz, 4H, H_Ar_), 7.36-7.20 (m, 10H, H_Ar_), 7.09 (t, *J* = 7.2 Hz, 1H, CH) (Fig. S5); ^13^C NMR (125 MHZ, DMSO-*d_6_*) *δ* (ppm): 165.95, 139.54, 134.09, 132.31, 131.18, 129.06, 128.63, 128.00, 127.16, 59.75 (Fig. S6).

**Product 6a:** *N,N*′-(­4-Methylphenylmethylene)dibenzamide;^1^H NMR (500 MHZ, DMSO-*d_6_*) *δ* (ppm): 8.94 (d, *J* = 7.7 Hz, 2H, 2NH), 7.96 (d, *J* = 8.0 Hz, 4H, H_Ar_), 7.54-7.46 (m, 6H, H_Ar_), 7.35 (d, *J* = 7.7 Hz, 2H, H_Ar_), 7.21 (d, *J* = 7.7 Hz, 2H, H_Ar_), 7.01 (t, *J* = 7.1 Hz, 1H, CH), 2.29 (s, 3H, CH_3_) (Fig. S7); ^13^C NMR (125 MHZ, DMSO-*d_6_*) *δ* (ppm): 166.22, 137.77, 136.82, 134.01, 131.98, 129.68, 128.16, 127.53, 126.25, 58.02, 20.62 (Fig. S8).

**Product 10a:** *N,N*′-(4-Nitrophenylmethylene)diacetamide;^1^H NMR (500 MHz, DMSO-*d*_6_) δ (ppm): 8.74 (d, *J* = 7.9 Hz, 2H, 2NH), 8.24 (d, *J* = 8.8 Hz, 2H, H_Ar_), 7.60 (d, *J* = 8.8 Hz, 2H, H_Ar_), 6.56 (t, *J* = 7.6 Hz, 1H, CH), 1.90 (s, 6H, 2CH_3_) (Fig. 9); ^13^C NMR (125 MHz, DMSO-*d*_6_) δ (ppm)­: 169.56, 148.43, 147.58, 128.35, 124.06, 57.60, 23.08 (Fig. 10).

**Product 11a:** *N,N*′-(4-Methoxyphenylmethylene)diacetamide;^1^H NMR (500 MHz, DMSO-*d*_6_) δ (ppm): 8.46 (d, *J* = 7.9 Hz, 2H, 2NH), 7.23 (d, *J* = 8.7 Hz, 2H, H_Ar_), 6.92 (d, *J* = 8.7 Hz, 2H, H_Ar_), 6.46 (t, *J* = 7.8 Hz, 1H, CH), 3.74 (s, 3H, OCH_3_), 1.85 (s, 6H, 2CH_3_) (Fig. 11); ^13^C NMR (125 MHz, DMSO-*d*_6_) δ (ppm)­: 169.03, 159.31, 133.18, 128.17, 114.18, 57.47, 55.78, 23.11 (Fig. 12).

**Product 13a:** *N,N*′-(4-Chlorophenylmethylene)­diacetamide;^1^H NMR (500 MHZ, DMSO-*d_6_*) *δ* (ppm): 8.57 (d, *J* = 7.8 Hz, 2H, 2NH), 7.43 (d, *J* = 8.5 Hz, 2H, H_Ar_), 7.33 (d, *J* = 8.5 Hz, 2H, H_Ar_), 6.47 (t, *J* = 7.8 Hz, 1H, CH), 1.86 (s, 6H, 2CH_3_) (Fig. 13). ^13^C NMR (125 MHZ, DMSO-*d_6_*) *δ* (ppm): 169.27, 140.10, 132.71, 128.92, 128.80, 57.41, 23.08 (Fig. S14).

**Selected spectroscopic data of synthesized derivatives of SMC reaction**

**Product 1b:** Biphenyl; ^1^H NMR (500 MHz; CDCl_3_) *δ* (ppm): 7.66 (d, *J* = 8.0 Hz, 4H)_,_ 7.50 (t, *J* = 7.8 Hz, 4H), 7.42 (t, *J* = 7.8 Hz, 2H) (Fig. S15). ^13^C NMR (125 MHz; CDCl_3_) *δ* (ppm):141.29, 128.81, 127.31, 127.23 (Fig. S16).

**Product 2b:** 4-Methylbiphenyl; ^1^H NMR (500 MHz; CDCl_3_) δ­: 7.70 (d, *J* = 7.5 Hz, 2H, H_Ar_), 7.61 (t, *J* = 7.0 Hz, 2H, H_Ar_), 7.54 (t, *J* = 7.5 Hz, 2H, H_Ar_), 7.44 (t, *J* = 7.5 Hz, 1H, H_Ar_), 7.36 (t, *J* = 7.0 Hz, 2H, H_Ar_), 2.51 (s, 3H) (Fig. S17); ­^13^CNMR (125 MHz; CDCl_3_) δ: 141.27, 138.47, 136.79, 129.60, 128.83, 127.28, 127.09, 126.92, 21.21 (Fig. S18).

**Product 4b:** 4-Methoxy-1,1'-biphenyl; ^1^H NMR (500 MHz; CDCl_3_) *δ* (ppm): 7.60 (t, *J* = 8.9 Hz, 4H, H_Ar_), 7.47 (t, *J* = 8.1 Hz, 2H, H_Ar_), 7.36 (t, *J* = 7.4 Hz, 1H, H_Ar_), 7.03 (d, *J* = 8.5 Hz, 2H, H_Ar_), 3.92 (s, 3H) (Fig. S19); ^13^CNMR (125 MHz; CDCl_3_) δ: 159.19, 140.83, 133.82, 128.77, 128.23, 126.78, 126.72, 114.22, 55.35 (Fig. S20).

**Product 10b:** 4-Phenylbenzonitrile; ^1^H NMR (500 MHz; CDCl_3_) *δ* (ppm): 7.73 (d, *J* = 8.3 Hz, 2H, H_Ar_), 7.67 (d, *J* = 8.3 Hz, 2H, H_Ar_), 7.58 (d, *J* = 7.3 Hz, 2H, H_Ar_), 7.47 (t, *J* = 7.3 Hz, 2H, H_Ar_), 7.40 (t, *J* = 7.3 Hz, 1H, H_Ar_) (Fig. S21); ^13^CNMR (125 MHz; CDCl_3_) δ: 145.35, 139.41, 133.01, 129.27, 128.54, 127.83, 127.15, 119.08, 110.25 (Fig. S22).

**Product 11b:** 4-Nitro-biphenyl; ^1^H NMR (500 MHz; CDCl_3_) *δ* (ppm): 8.29 (d, *J* = 8.8 Hz, 2H, H_Ar_), 7.73 (d, *J* = 8.8 Hz, 2H, H_Ar_), 7.62 (d, *J* = 8.3 Hz, 2H, H_Ar_), 7.50-7.41 (m, 3H, H_Ar_) (Fig. S23); ^13^CNMR (125 MHz; CDCl_3_) δ: 147.02, 138.76, 134.91, 129.01, 128.26, 127.46, 127.00, 124.35 (Fig. S24).

**Product 14b:** 4-Acetylbiphenyl; ^1^H NMR (500 MHz; CDCl_3_) *δ* (ppm): 8.01 (d, *J* = 8.0 Hz, 2H, H_Ar_), 7.70 (d, *J* = 8.0 Hz, 2H, H_Ar_), 7.64 (d, *J* = 7.3 Hz, 2H, H_Ar_), 7.48 (t, *J* = 7.3 Hz, 2H, H_Ar_), 7.39 (t, *J* = 7.3 Hz, 1H, H_Ar_), 2.65 (s, 3H, CH_3_) (Fig. S25); ^13^CNMR (125 MHz; CDCl_3_) δ: 197.70, 145.05, 139.66, 136.17, 129.77, 129.55, 128.65, 127.91, 127.05, 27.92 (Fig. S26).

**Product 17b:** 4-Chloro-1,1'-biphenyl; ^1^H NMR (500 MHz; CDCl_3_) *δ* (ppm): 7.45 (d, *J* = 8.0 Hz, 2H, H_Ar_), 7.41 (d, *J* = 8.0 Hz, 2H, H_Ar_), 7.26-7.21 (m, 5H, H_Ar_) (Fig. S27); ^13^CNMR (125 MHz; CDCl_3_) δ: 140.63, 139.28, 133.43, 129.39, 128.81, 128.12, 127.23, 126.76 (Fig. S28).

**Product 23b:** 4-Phenylpyridine; ^1^H NMR (500 MHz; CDCl_3_) *δ* (ppm): 8.65 (d, *J* = 6.1 Hz, 2H, H_Ar_), 7.66-7.62 (m, 2H, H_Ar_), 7.50-7.43 (m, 5H, H_Ar_) (Fig. S29); ^13^CNMR (125 MHz; CDCl_3_) δ: 149.68, 148.03, 137.85, 129.65, 128.48, 126.43, 121.36 (Fig. S30).

**Product 24b:** 6-Phenylpyridin-3-amine; ^1^H NMR (500 MHz; CDCl_3_) *δ* (ppm): 8.16 (s, 1H, H_Ar_), 7.92 (d, *J* = 8.1 Hz, 2H, H_Ar_), 7.63 (d, *J* = 8.5 Hz, 1H, H_Ar_), 7.37 (t, *J* = 8.1 Hz, 2H, H_Ar_), 7.28 (t, *J* = 8.1 Hz, 1H, H_Ar_), 7.08 (d, *J* = 8.5 Hz, 1H, H_Ar_), 5.21 (s, 2H, NH_2_) (Fig. S31); ^13^CNMR (125 MHz; CDCl_3_) δ: 144.69, 143.54, 139.95, 137.18, 129.33, 127.43, 125.43, 121.06, 120.27 (Fig. S32).

**Product 25b:** 7-Chloro-4-phenylquinoline; ^1^H NMR (500 MHz; CDCl_3_) *δ* (ppm): 8.92 (d, *J* = 4.6 Hz, 1H, H_Ar_), 8.14 (d, *J* = 2.3 Hz, 1H, H_Ar_), 7.88 (d, *J* = 9.0 Hz, 1H, H_Ar_), 7.60-7.49 (m, 6H, H_Ar_), 7.34 (d, *J* = 4.6 Hz, 1H, H_Ar_) (Fig. S33); ^13^CNMR (125 MHz; CDCl_3_) δ: 152.22, 149.83, 146.05, 138.26, 135.17, 131.29, 130.52, 129.63, 129.03, 128.76, 127.43, 125.42, 122.38 (Fig. S34).

**Product 26b:** 2-Chloro-4-phenylpyrimidine; ^1^H NMR (500 MHz; CDCl_3_) *δ* (ppm): 8.35 (d, *J* = 6.0 Hz, 1H, H_Ar_), 8.14-8.09 (m, 2H, H_Ar_), 7.66 (d, *J* = 6.0 Hz, 1H, H_Ar_), 7.56-7.49 (m, 3H, H_Ar_) (Fig. S35); ^13^CNMR (125 MHz; CDCl_3_) δ: 158.01, 162.07, 159.77, 136.06, 131.82, 129.79, 127.20, 115.98 (Fig. S36).

**Original spectrums of *N,N′*-alkylidene bisamides**

**
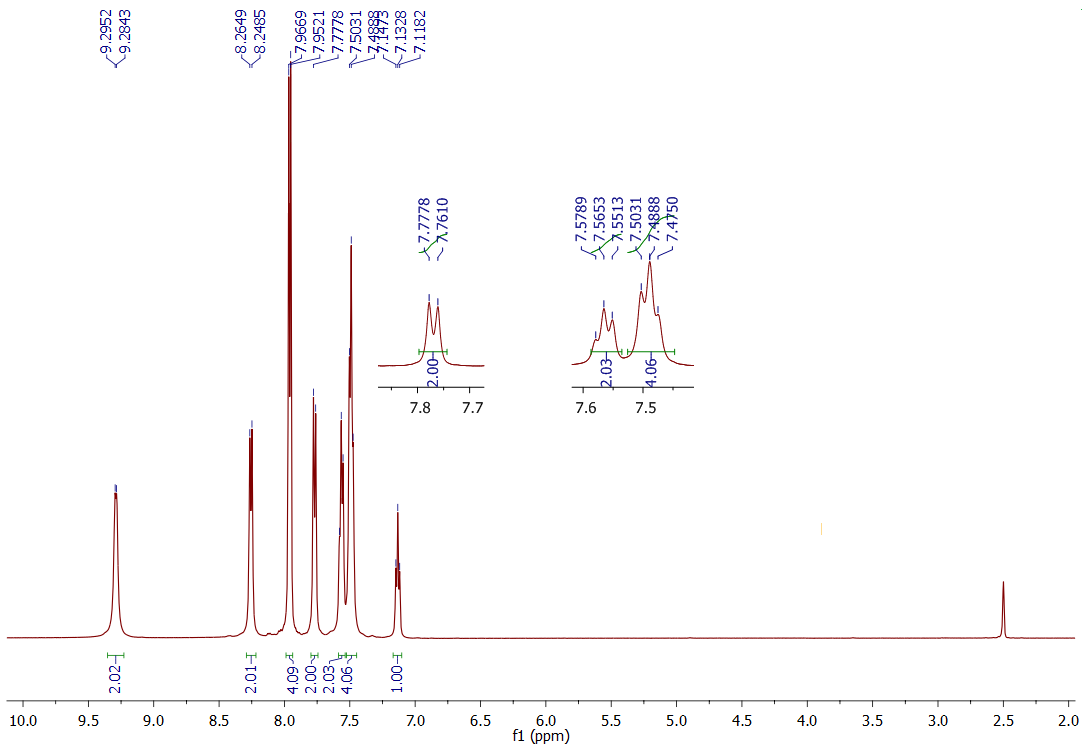
**

**Figure S1.** The ^1^H NMR spectrum of product **2a**

**
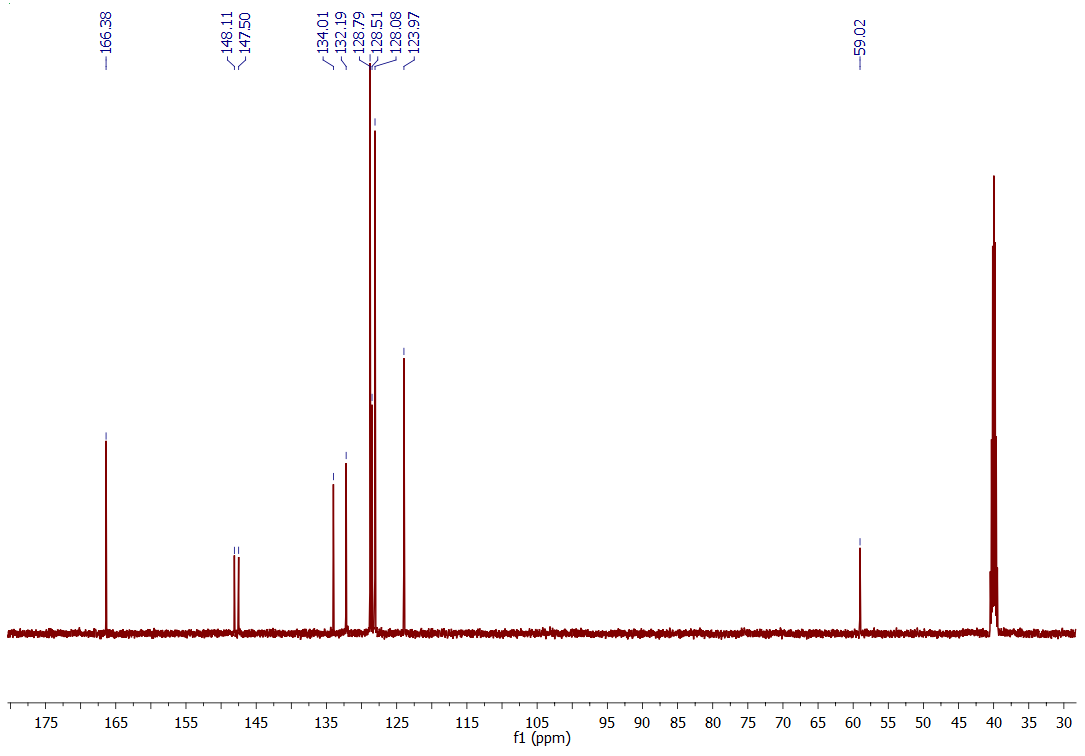
**

**Figure S2.** The ^13^C NMR spectrum of product **2a**

**
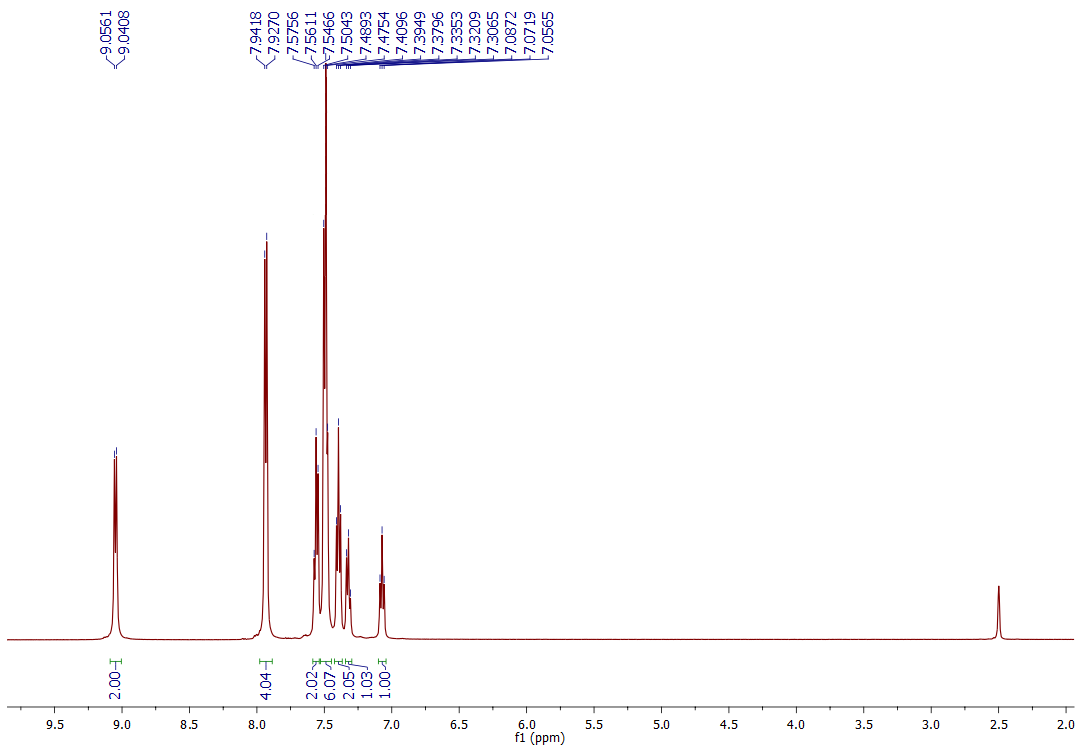
**

**Figure S3.** The ^1^H NMR spectrum of product **3a**

**
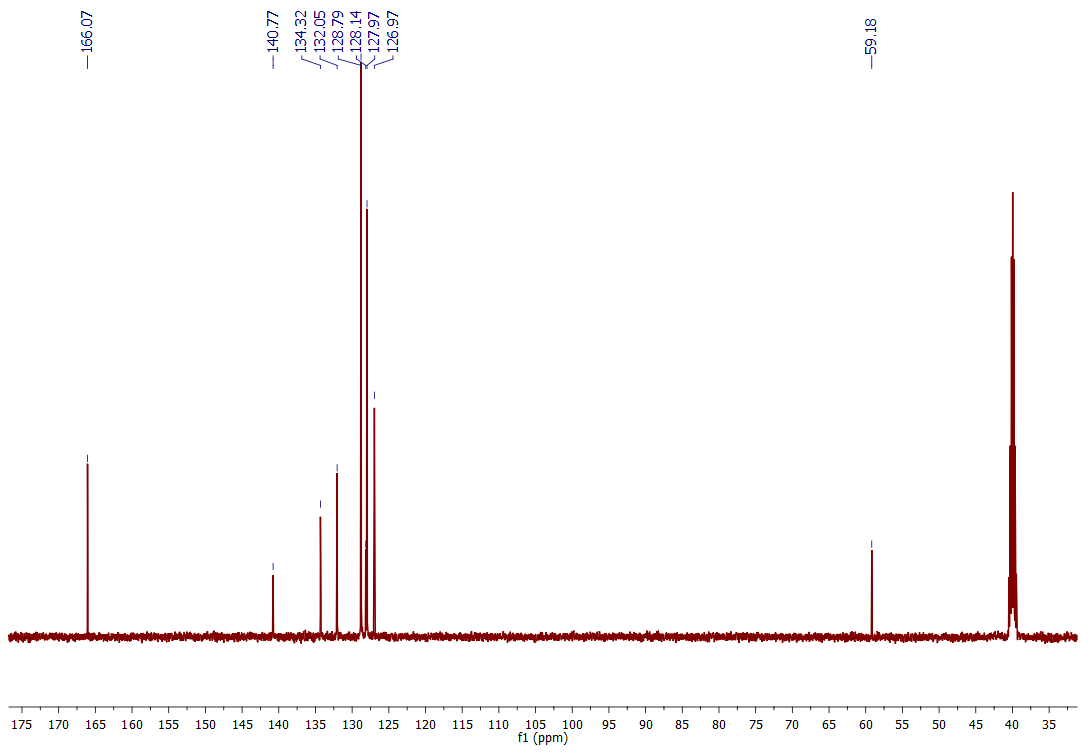
**

**Figure S4.** The ^13^C NMR spectrum of product **3a**


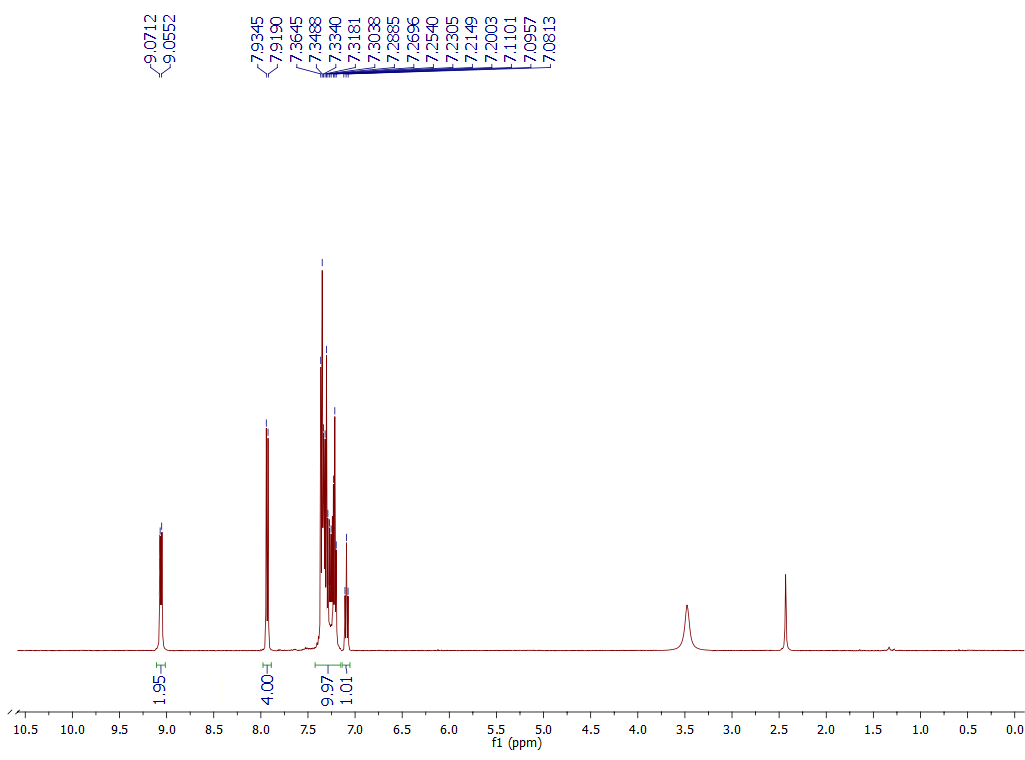

**Figure S5.** The ^1^H NMR spectrum of product **5a**

**
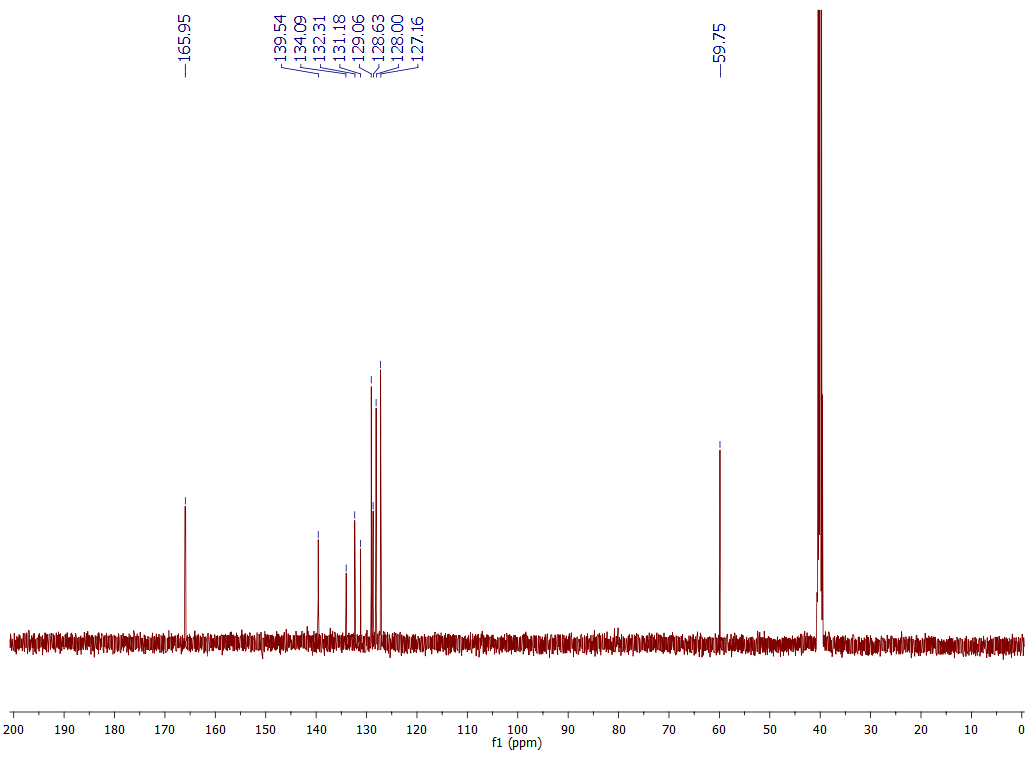
**

**Figure S6.** The ^13^C NMR spectrum of product **5a**


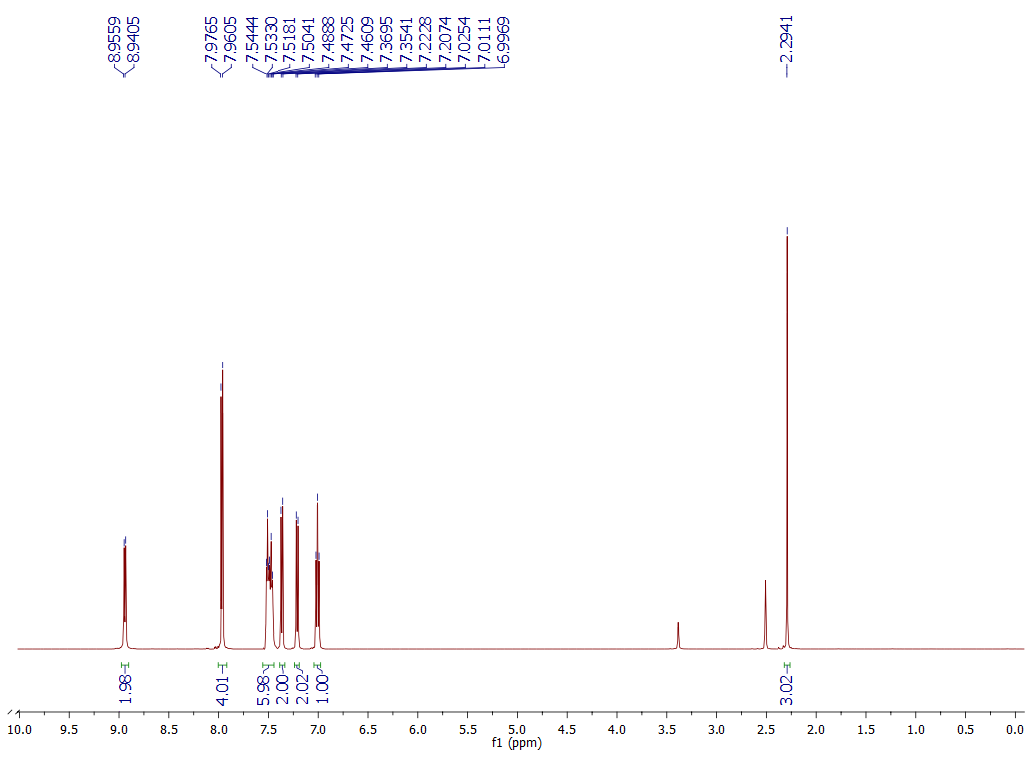

**Figure S7.** The ^1^H NMR spectrum of product **6a**


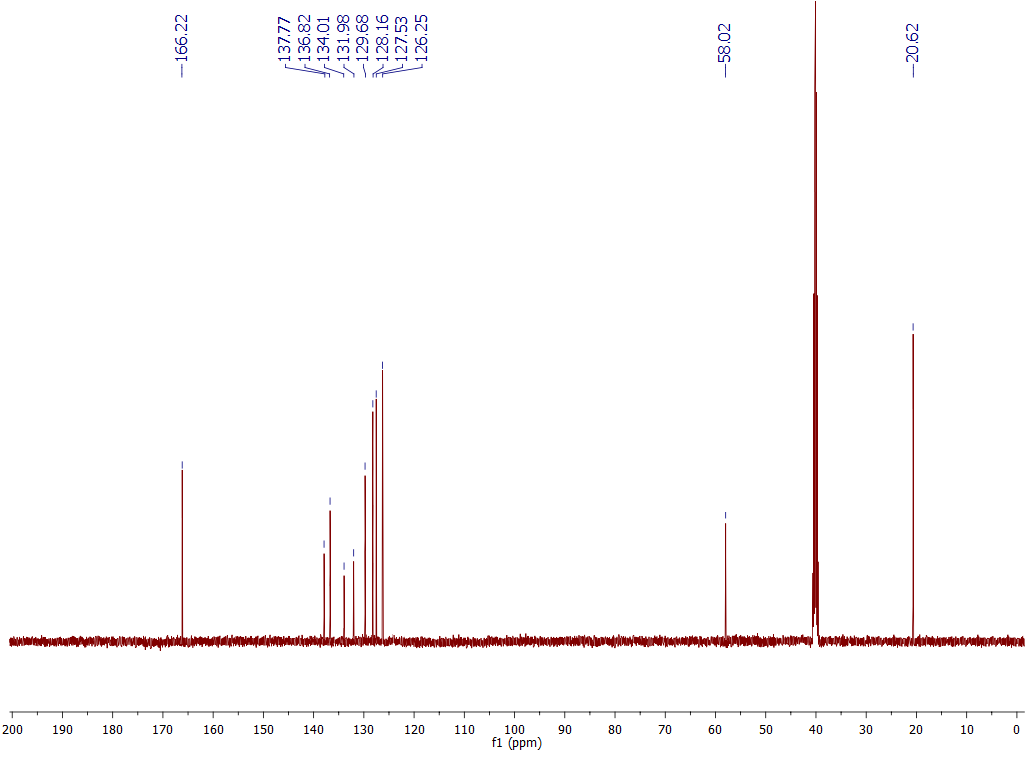

**Figure S8.** The ^13^C NMR spectrum of product **6a**


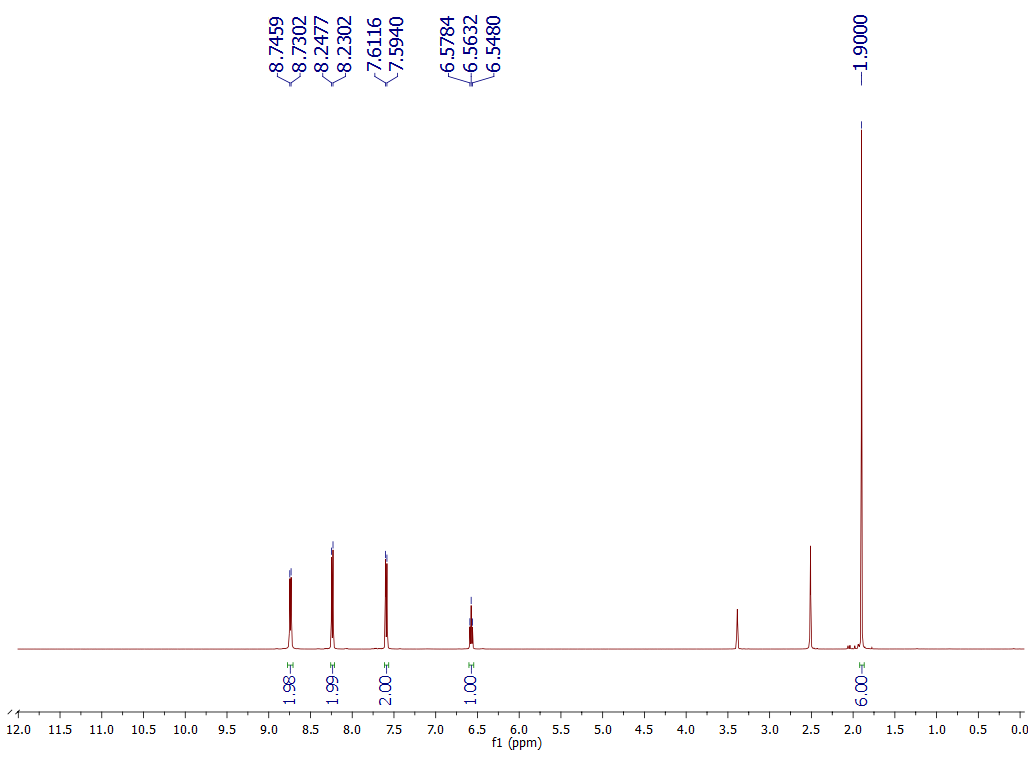

**Figure S9.** The ^1^H NMR spectrum of product **10a**

**
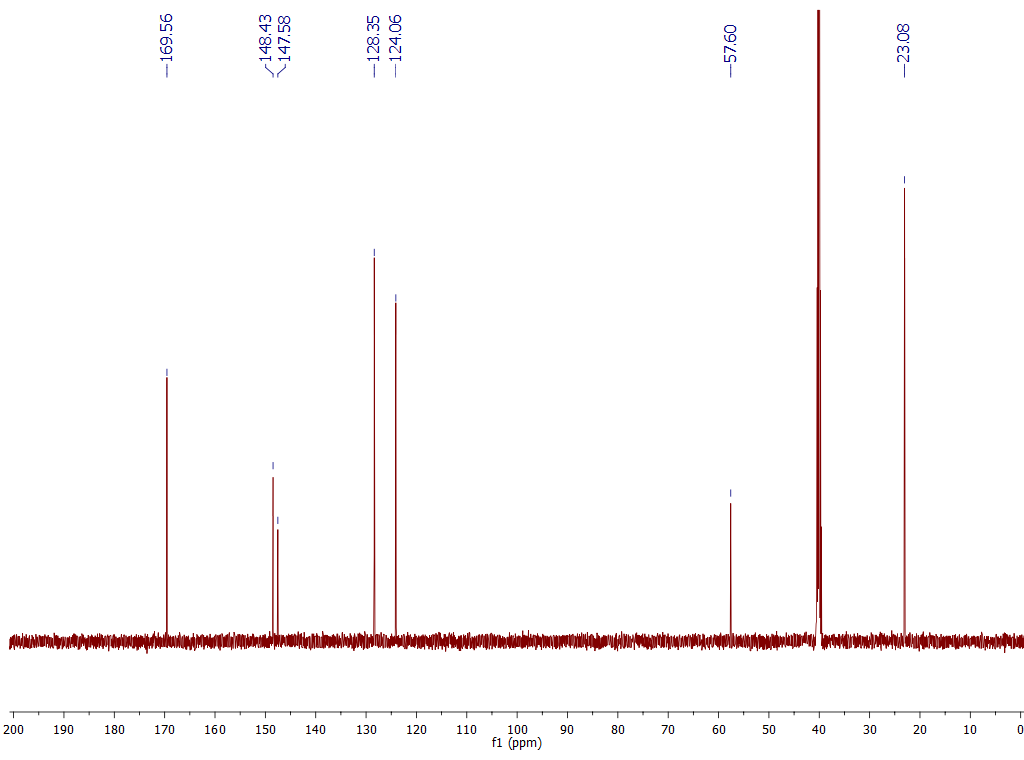
**

**Figure S10.** The ^13^C NMR spectrum of product **10a**


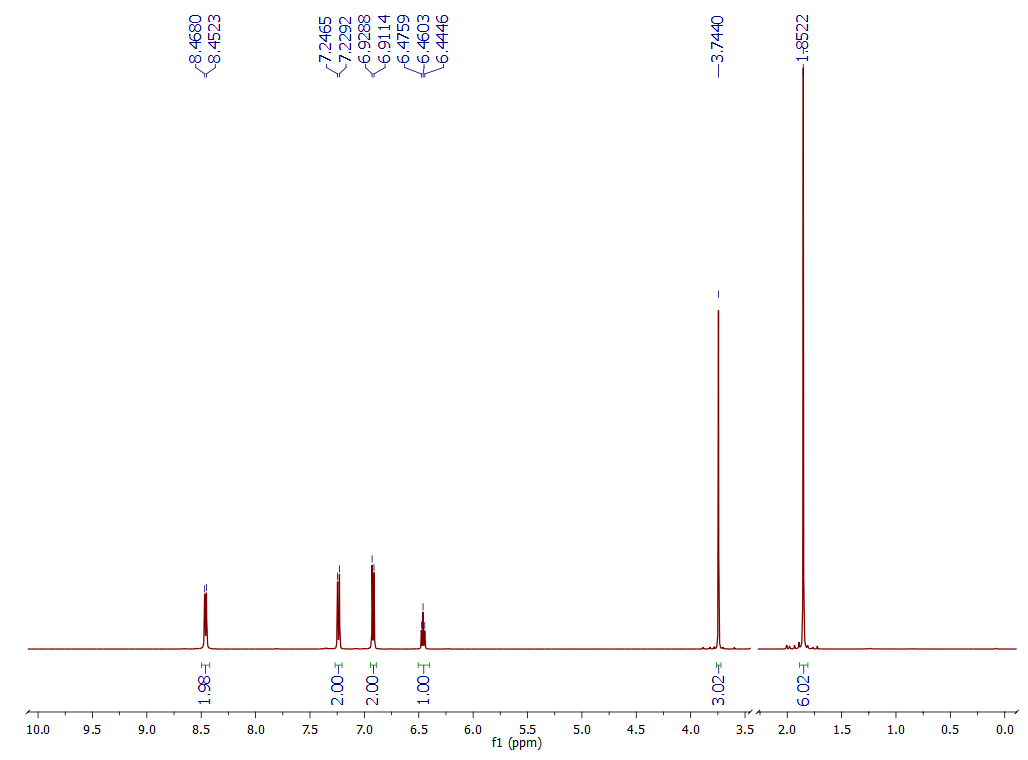

**Figure S11.** The ^1^H NMR spectrum of product **11a**


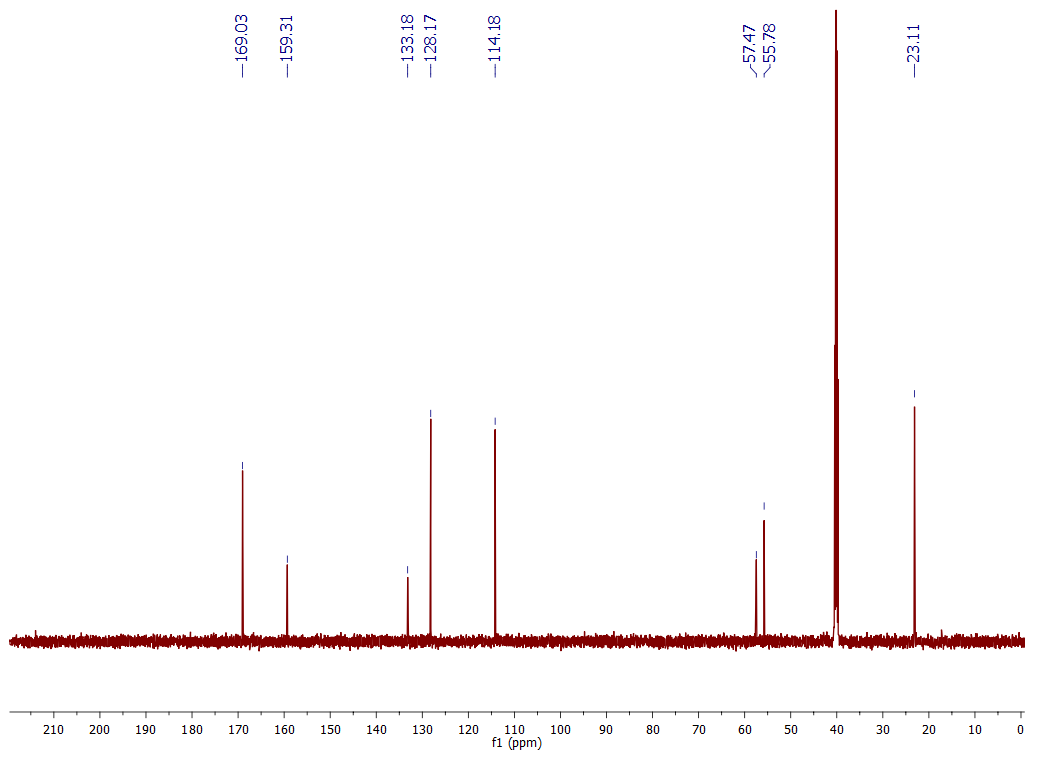

**Figure S12.** The ^13^C NMR spectrum of product **11a**


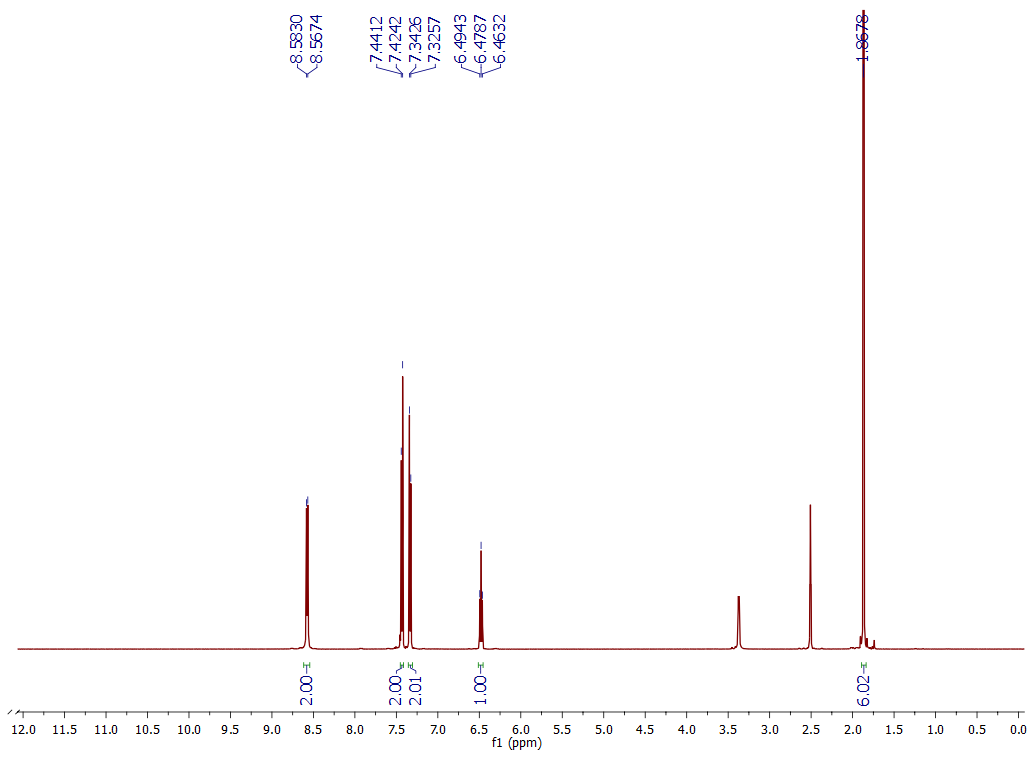

**Figure S13**. The ^1^H NMR spectrum of product **13a**


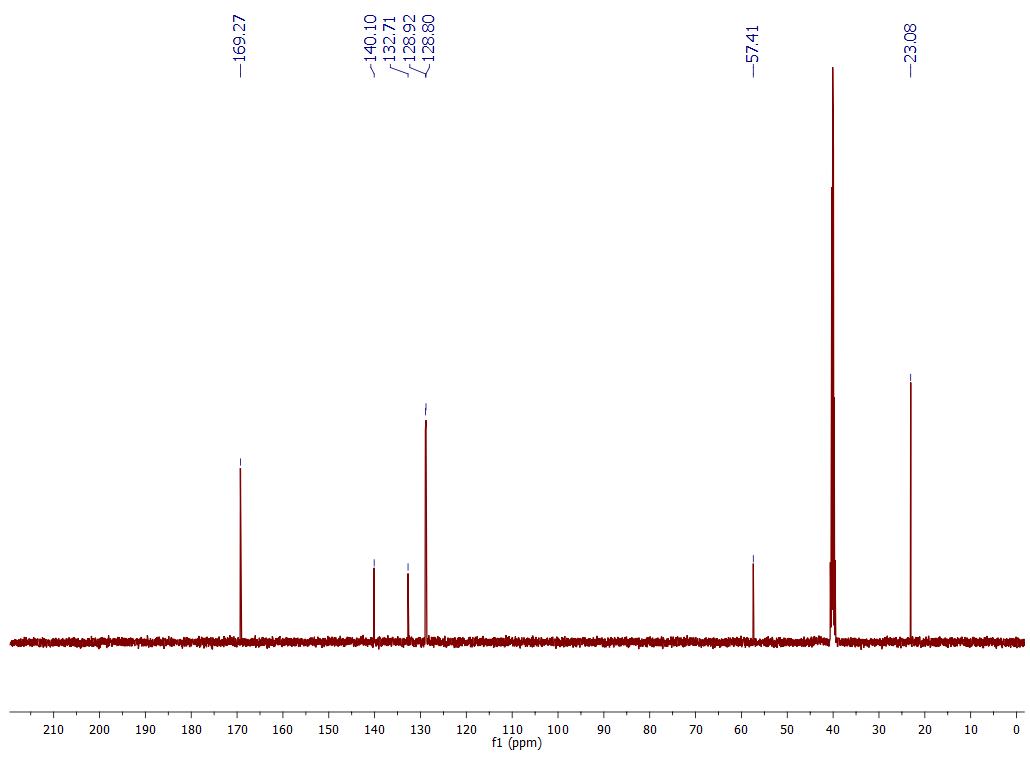

**Figure S14.** The ^13^C NMR spectrum of product **13a**

**Original spectrums of synthesized derivatives of SMC reaction**

**
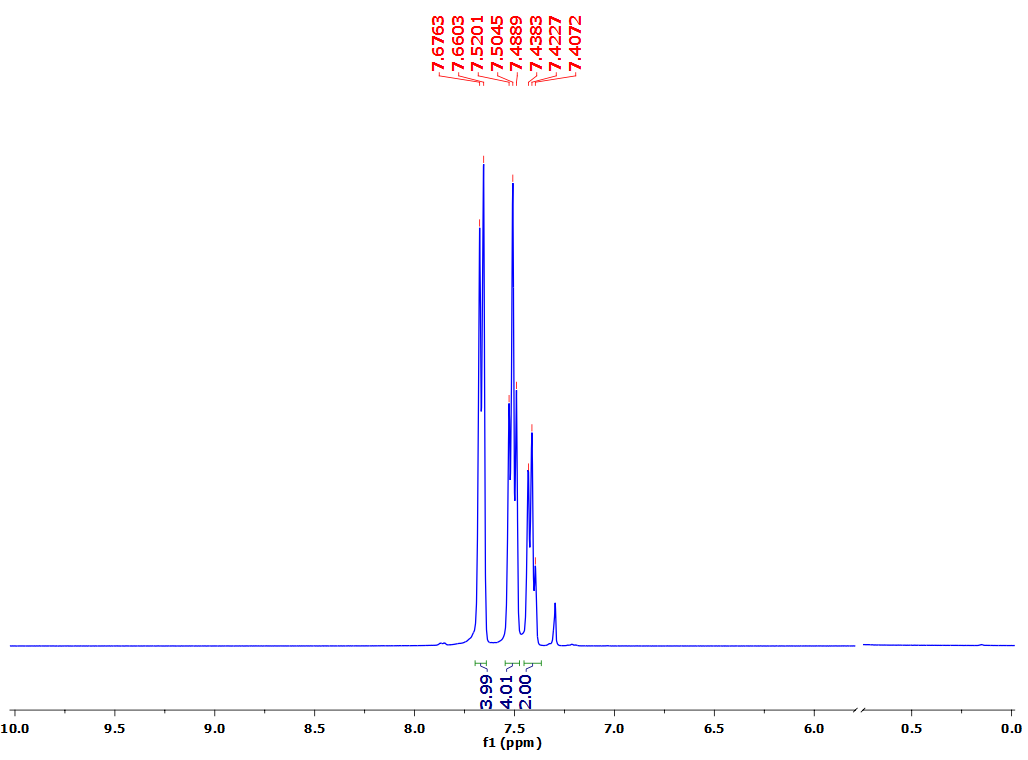
**

Figure S15. The ^1^H NMR spectrum of 1b


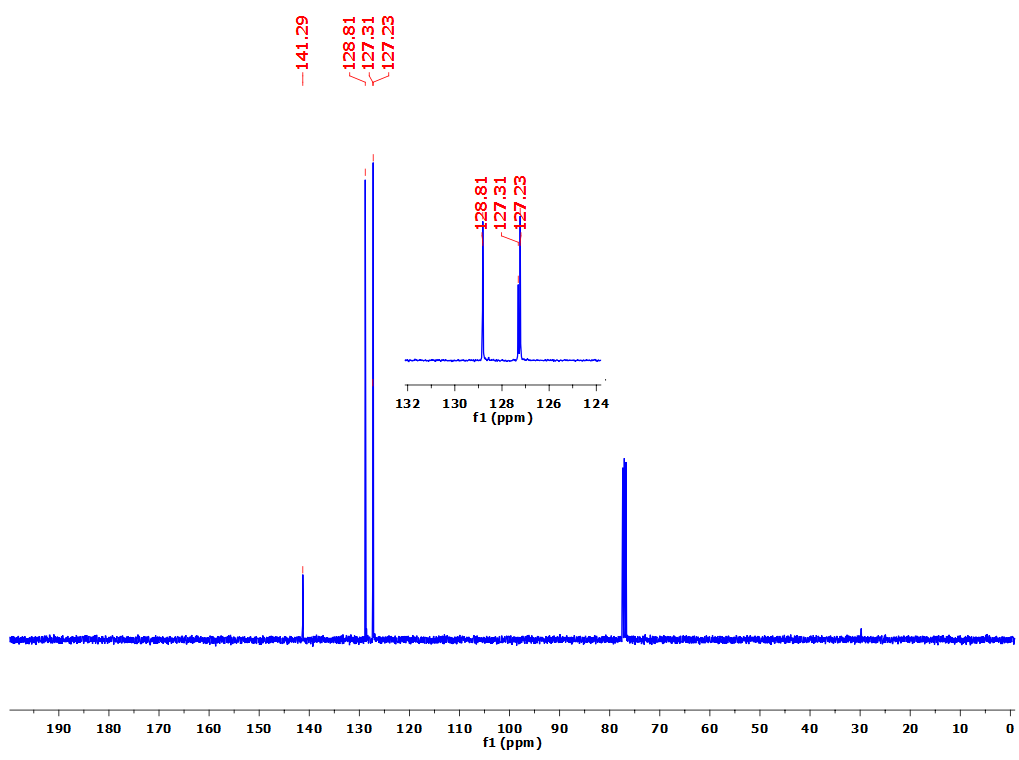

Figure S16. The ^1^H NMR spectrum of 1b

**
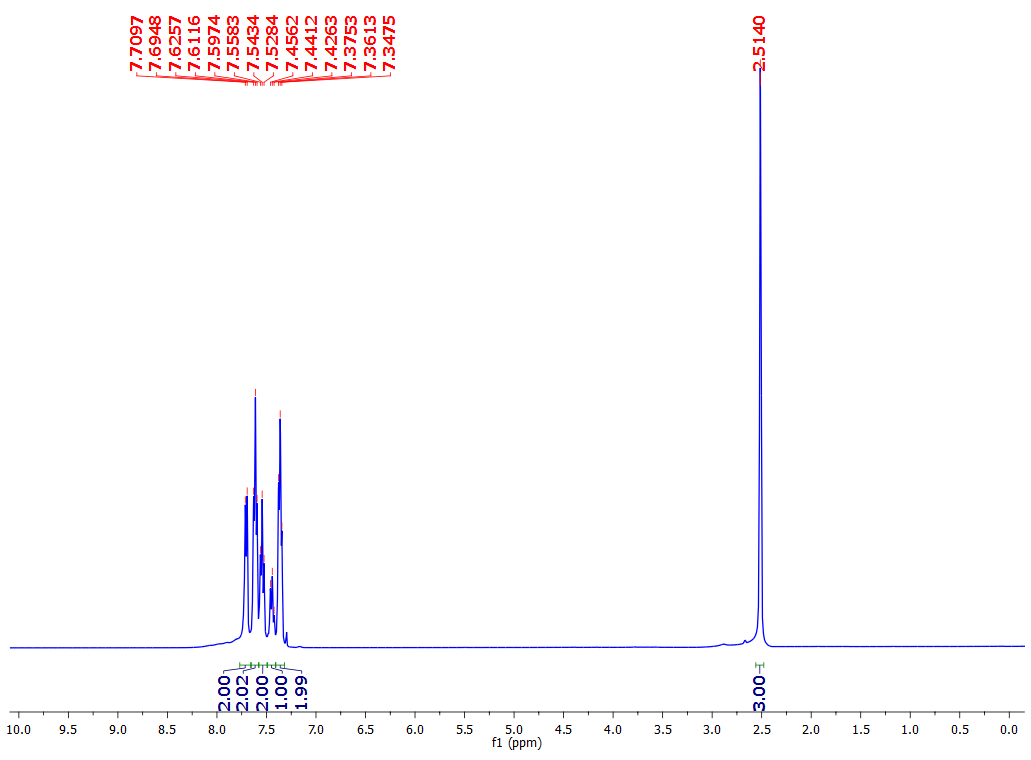
**

Figure S17. The ^1^H NMR spectrum of 2b

**
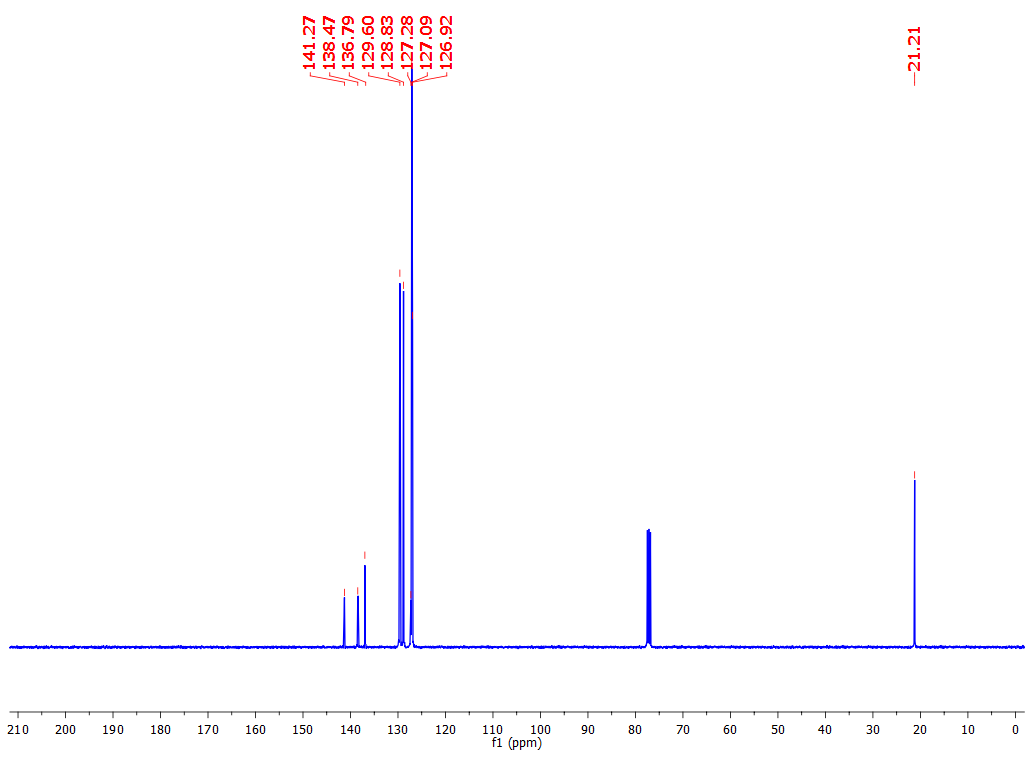
**

Figure S18. The ^13^C NMR spectrum of 2b


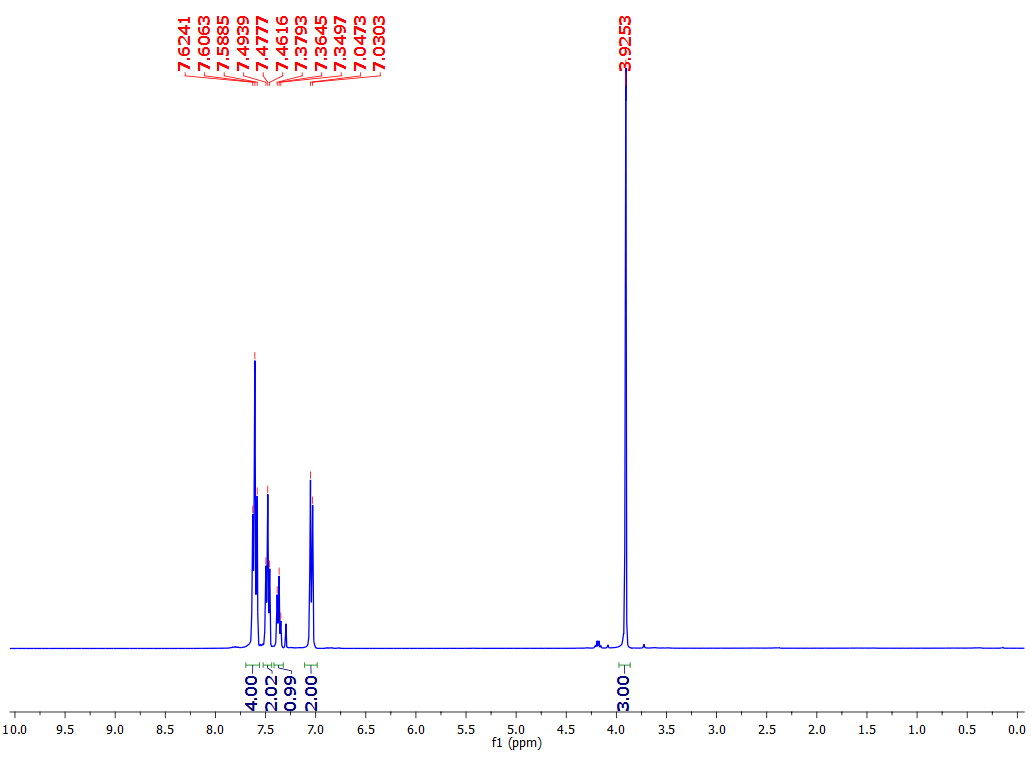

Figure S19. The ^1^H NMR spectrum of 4b

**
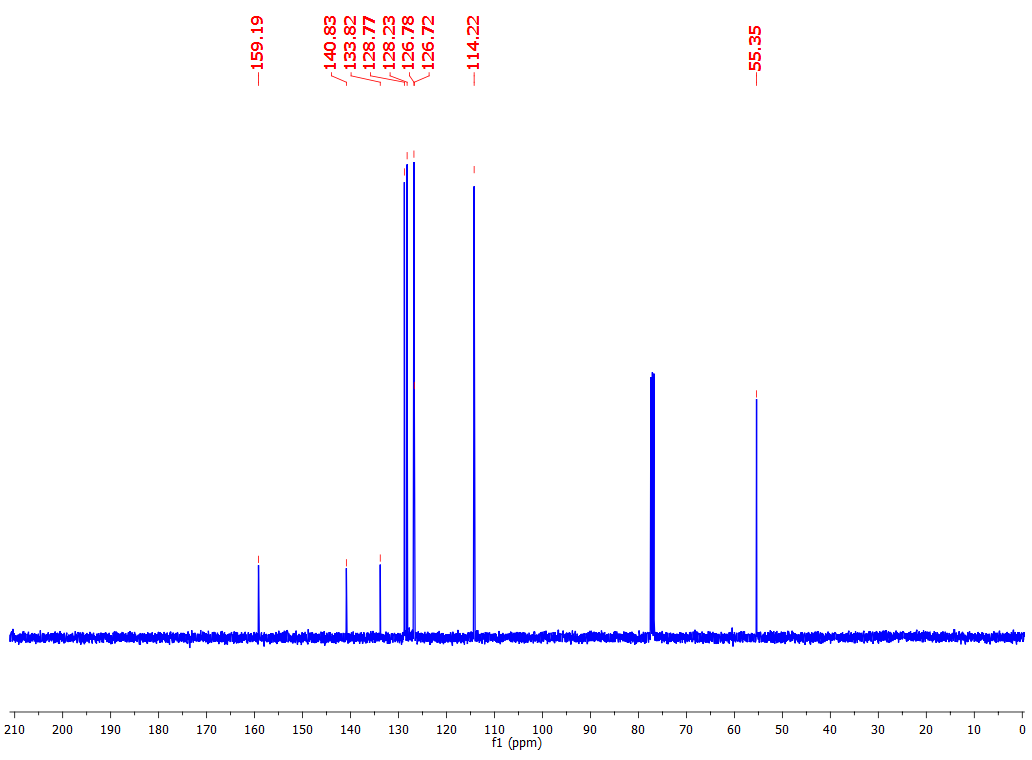
**

Figure S20. The ^13^C NMR spectrum of 4b


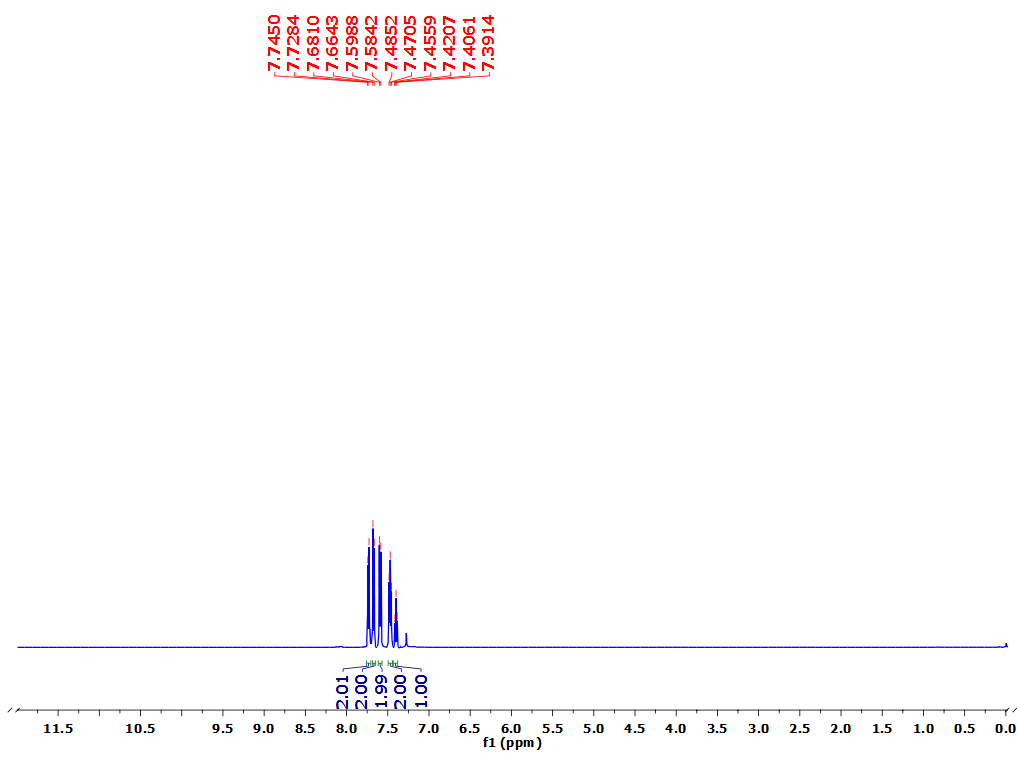

Figure S21. The ^1^H NMR spectrum of 10b


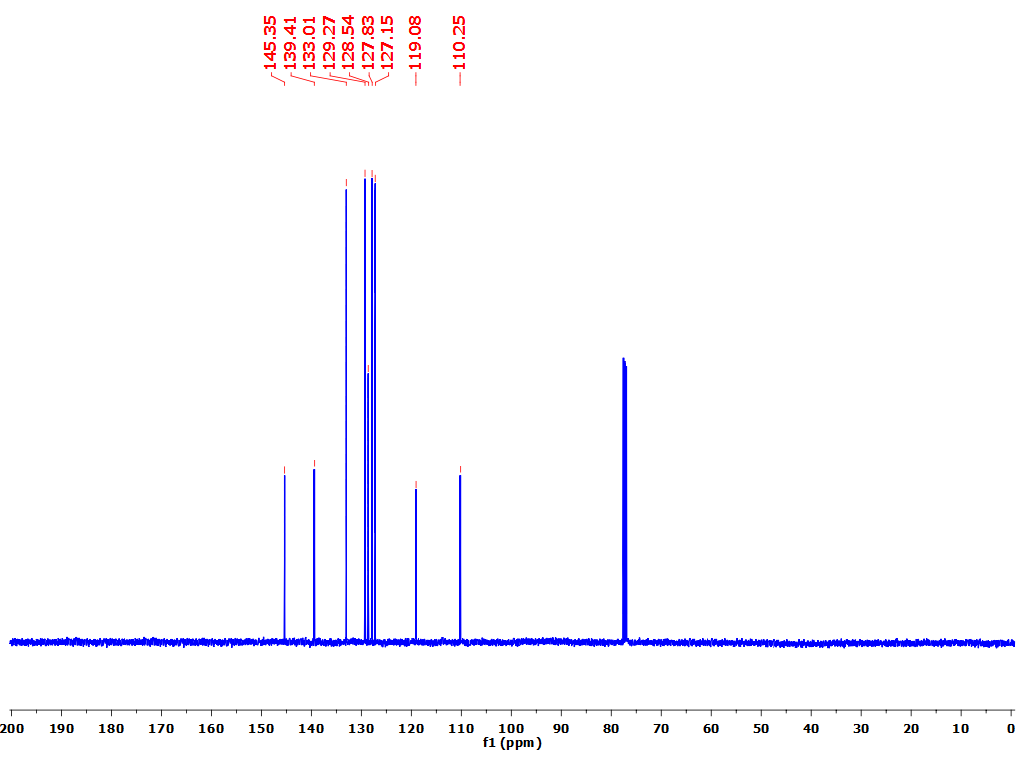

Figure S22. The ^13^C NMR spectrum of 10b


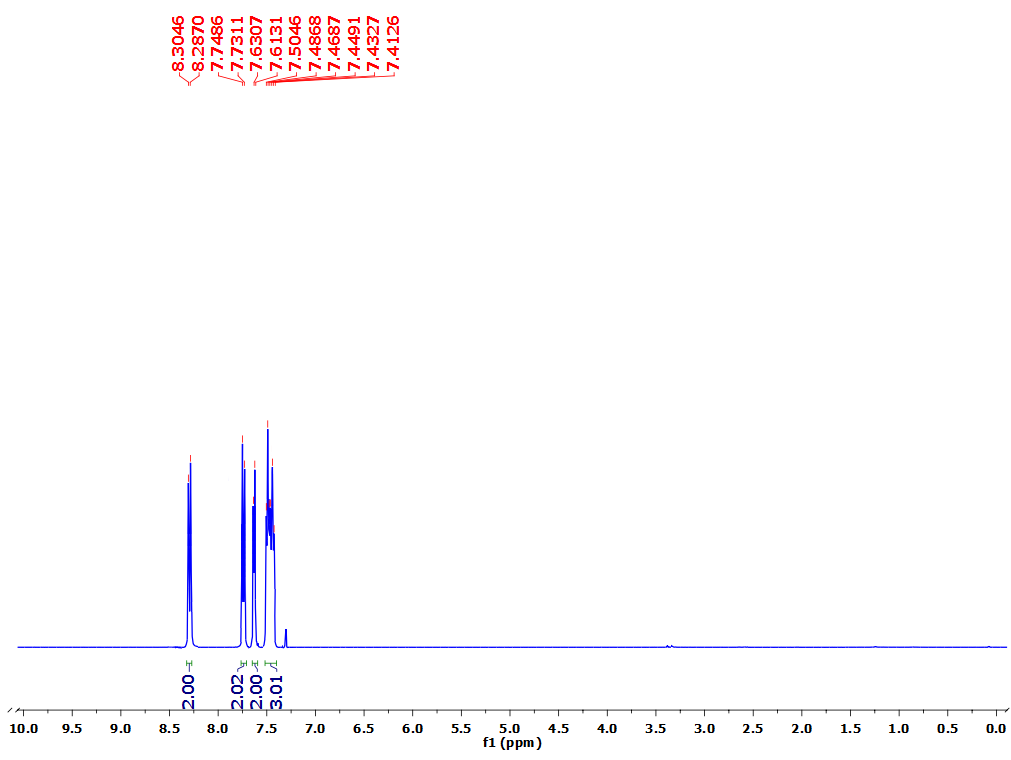

Figure S23. The ^1^H NMR spectrum of 11b


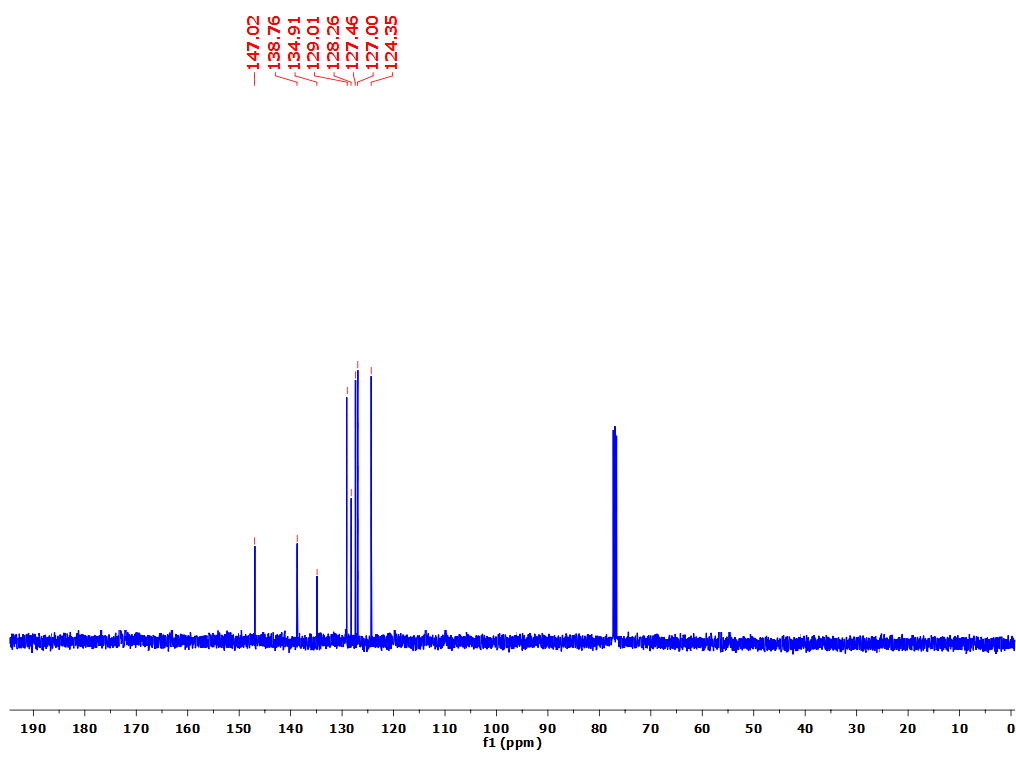

Figure S24. The ^13^C NMR spectrum of 11b


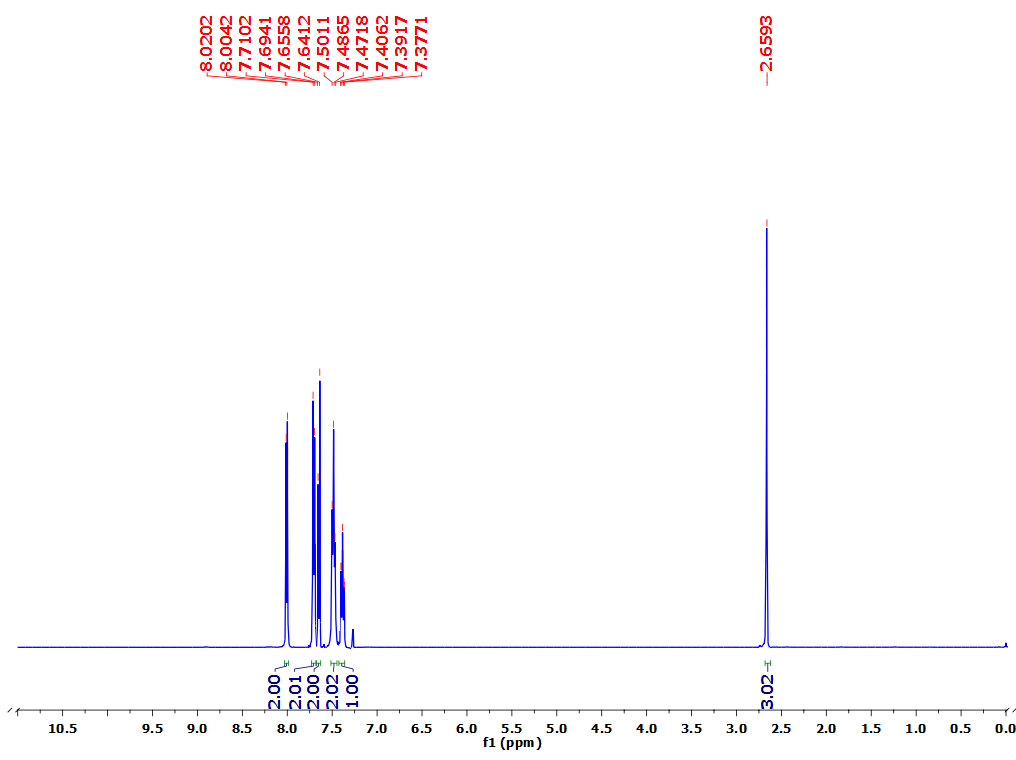

Figure S25. The ^1^H NMR spectrum of 14b


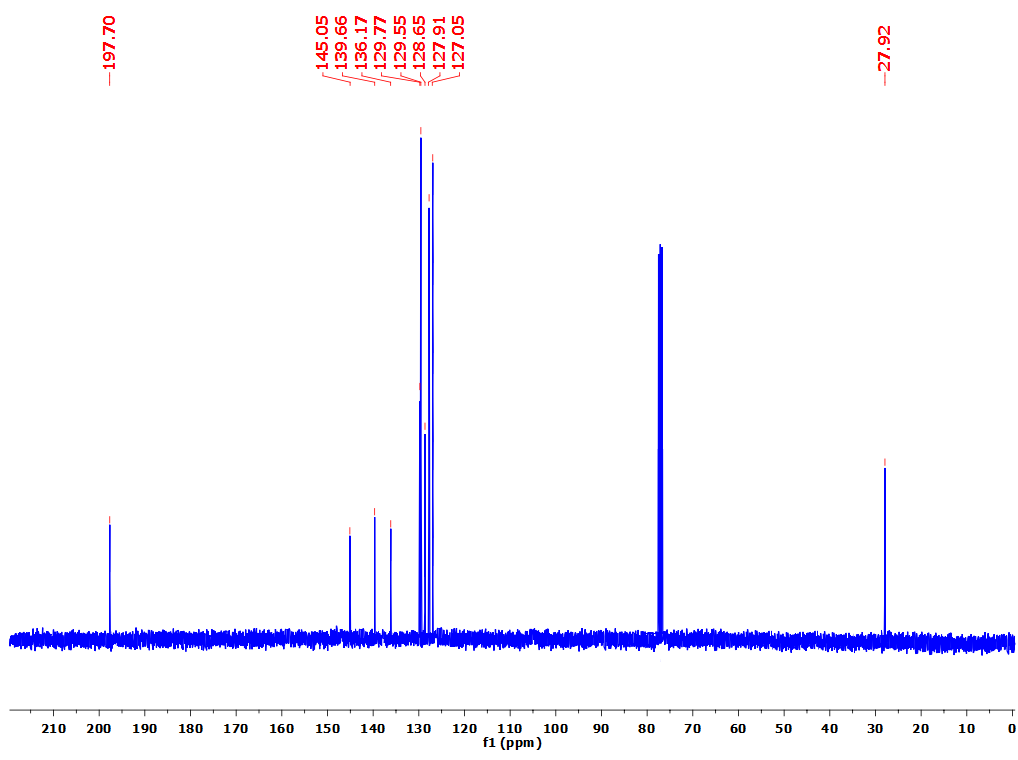

Figure S26. The ^13^C NMR spectrum of 14b


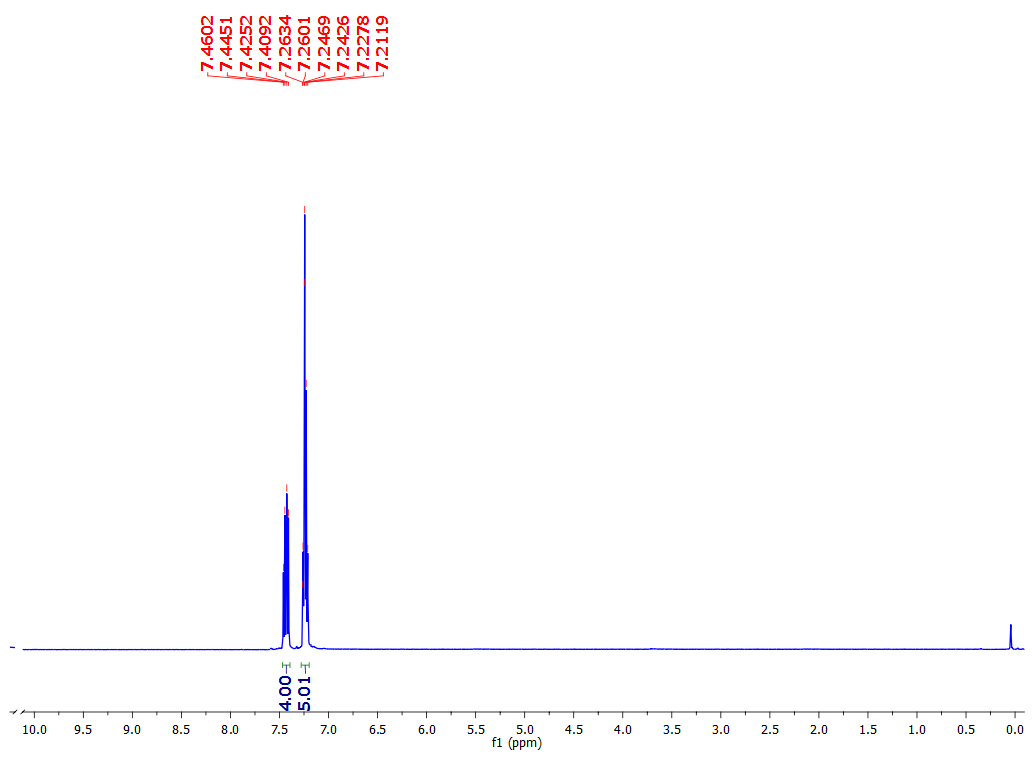

Figure S27. The ^1^H NMR spectrum of 17b


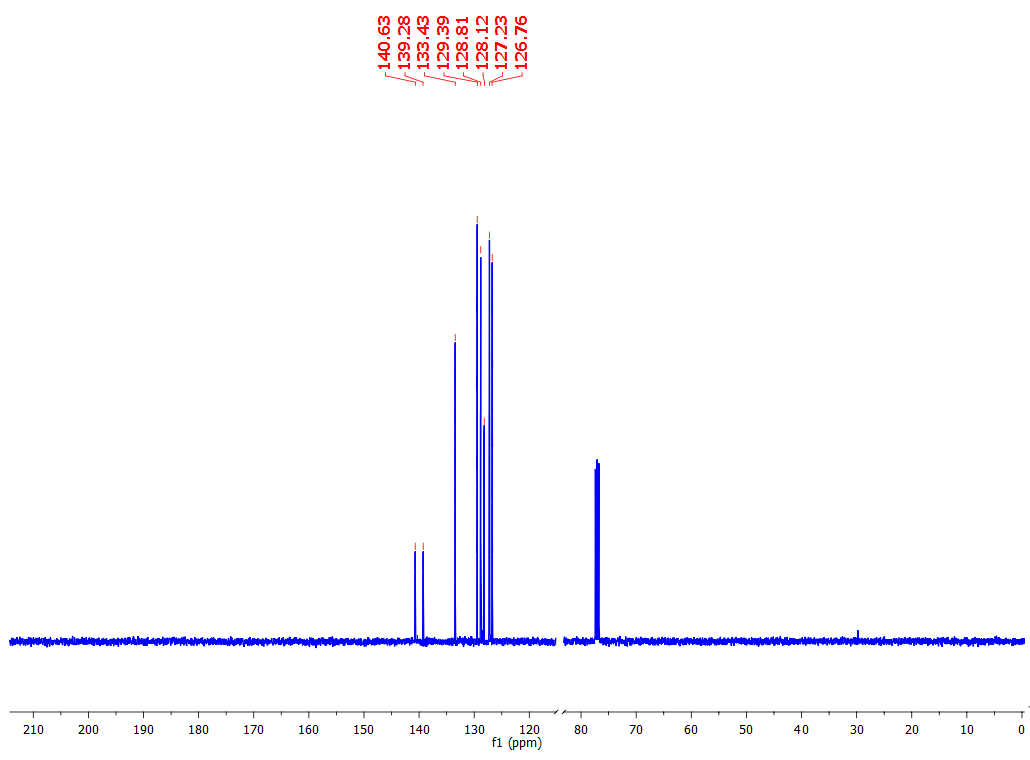

Figure S28. The ^13^C NMR spectrum of 17b


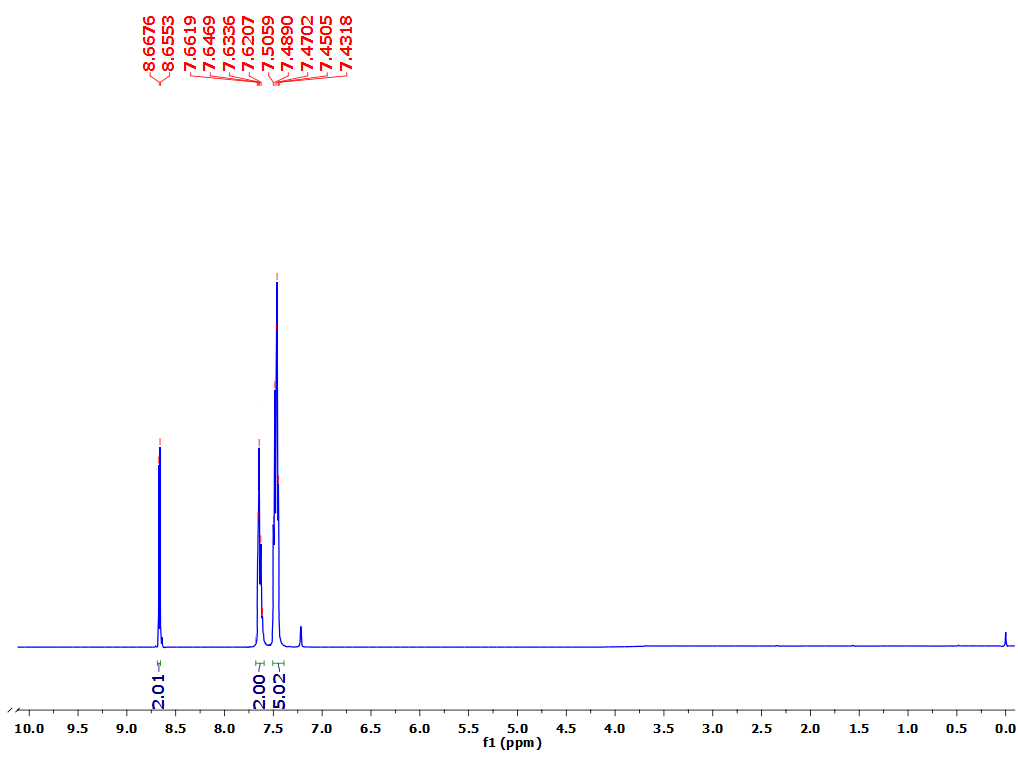

Figure S29. The ^1^H NMR spectrum of 23b


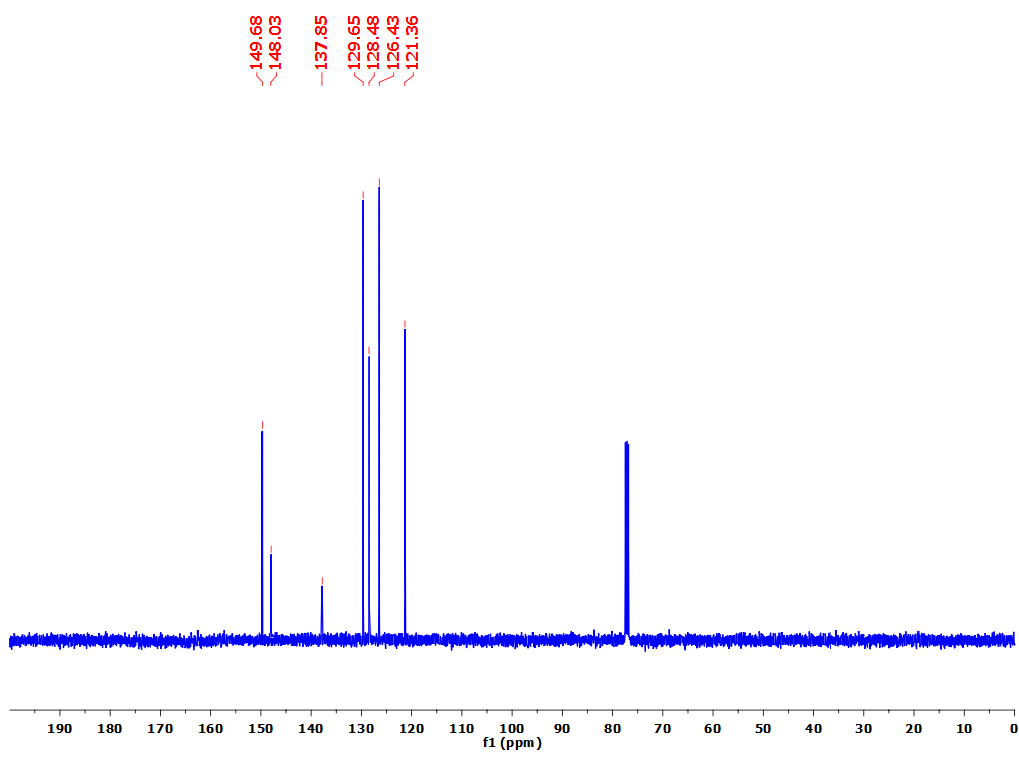

Figure S30. The ^13^C NMR spectrum of 23b


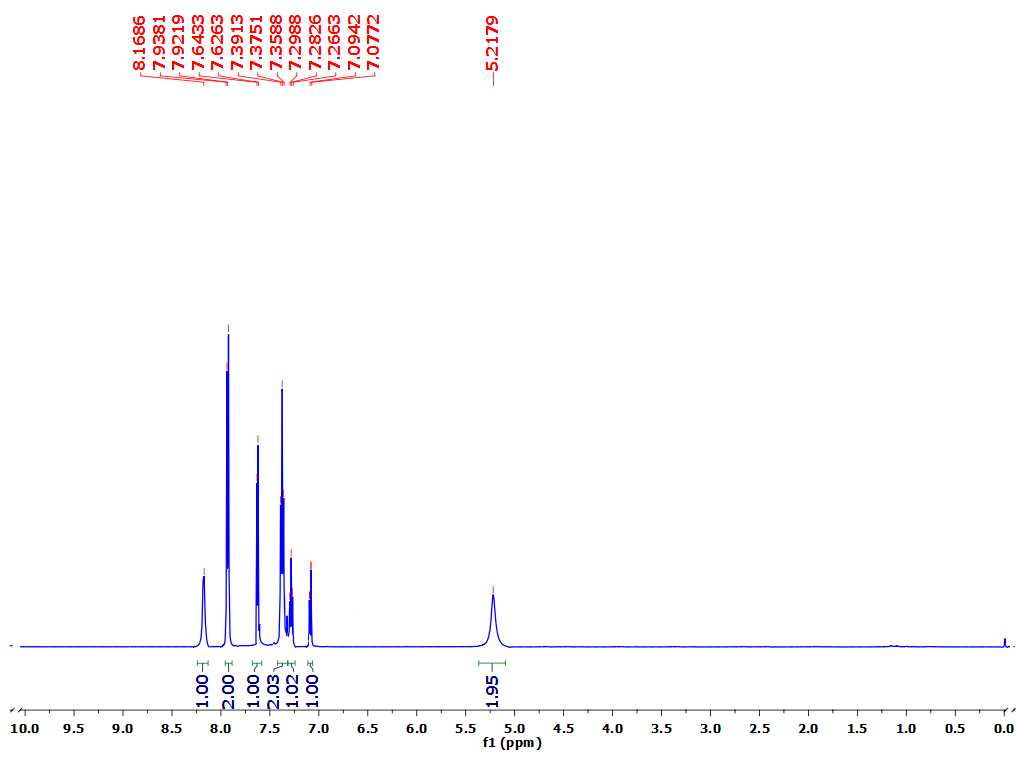

Figure S31. The ^1^H NMR spectrum of 24b


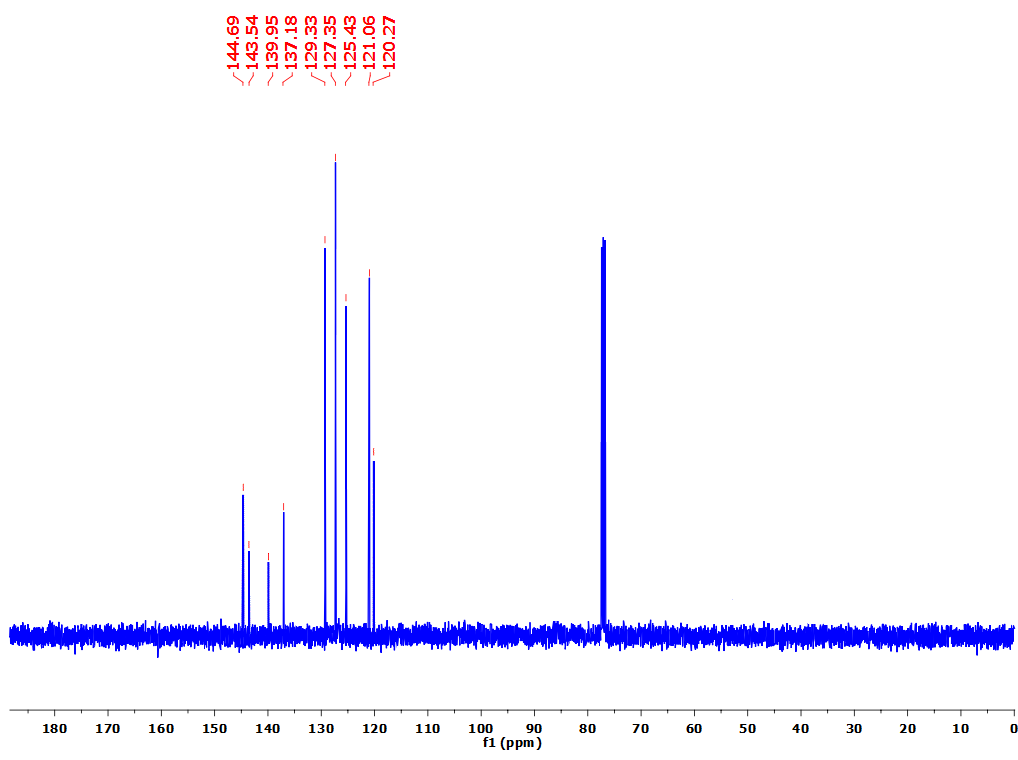

Figure S32. The ^13^C NMR spectrum of 24b


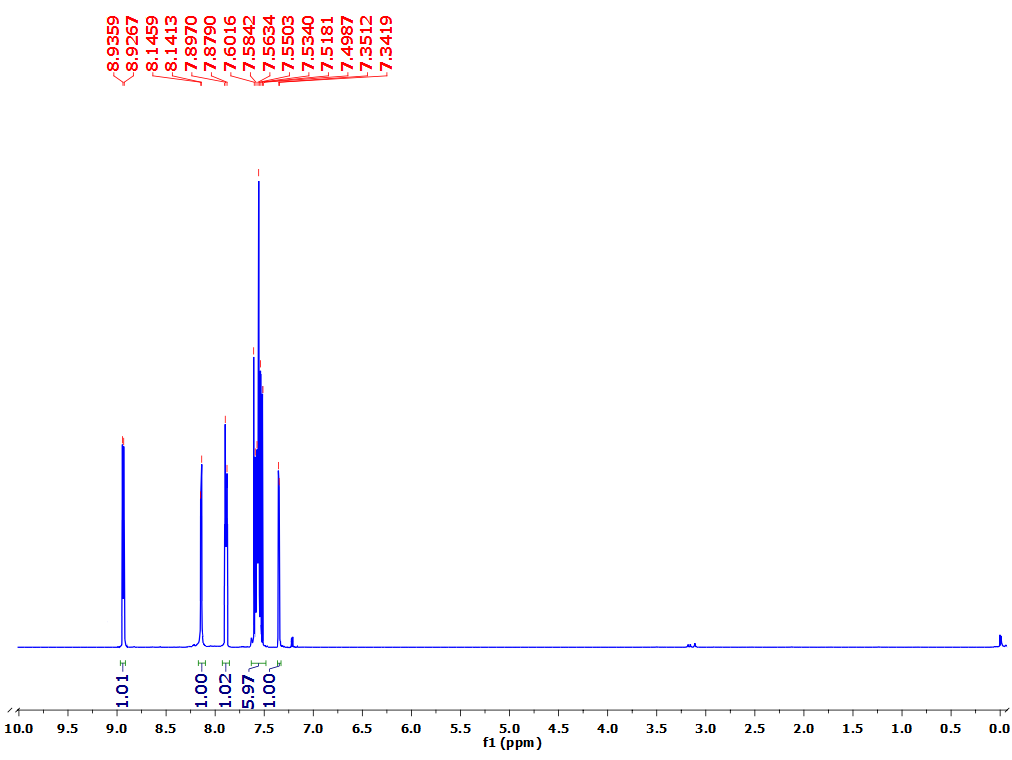

Figure S33. The ^1^H NMR spectrum of 25b


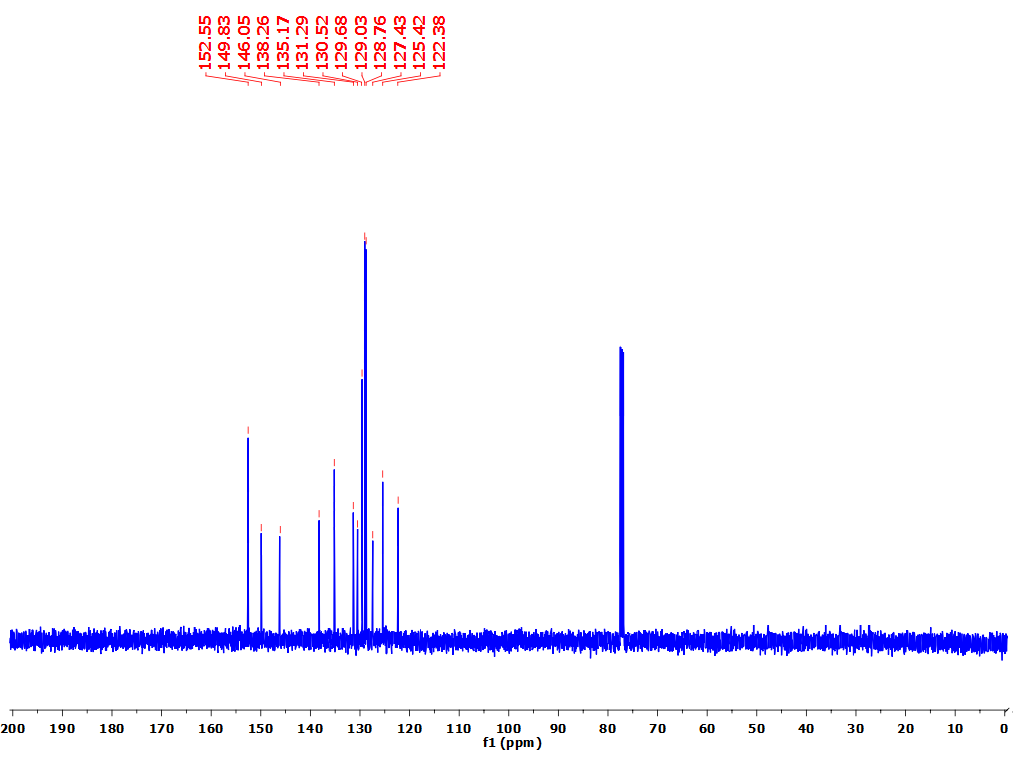

Figure S34. The ^13^C NMR spectrum of 25b


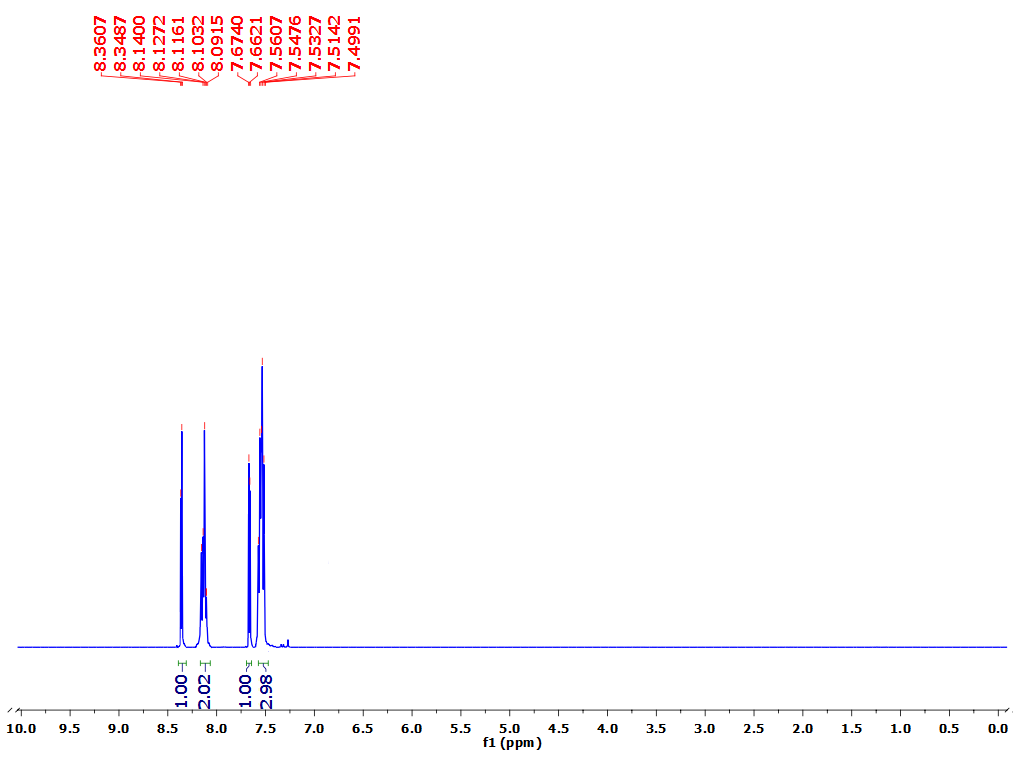

Figure S35. The ^1^H NMR spectrum of 26b


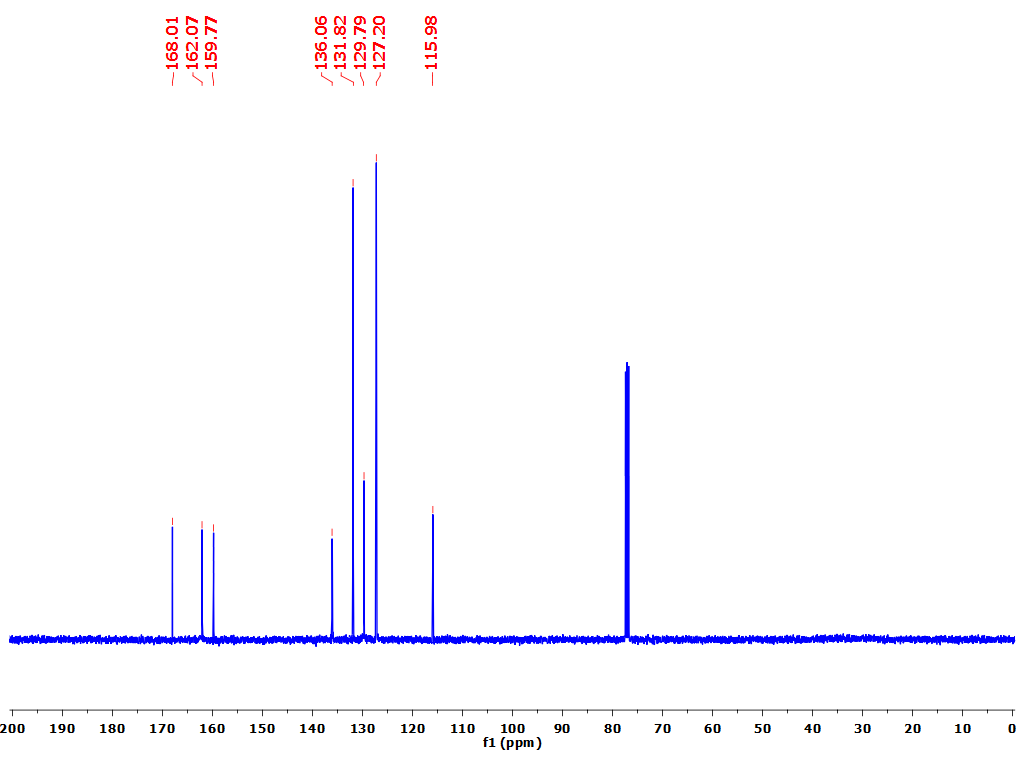

Figure S36. The ^13^C NMR spectrum of 26b
